# Integrated genome and transcriptome sequencing identifies a noncoding mutation in the genome replication factor *DONSON* as the cause of microcephaly-micromelia syndrome

**DOI:** 10.1101/gr.219899.116

**Published:** 2017-08

**Authors:** Gilad D. Evrony, Dwight R. Cordero, Jun Shen, Jennifer N. Partlow, Timothy W. Yu, Rachel E. Rodin, R. Sean Hill, Michael E. Coulter, Anh-Thu N. Lam, Divya Jayaraman, Dianne Gerrelli, Diana G. Diaz, Chloe Santos, Victoria Morrison, Antonella Galli, Ulrich Tschulena, Stefan Wiemann, M. Jocelyne Martel, Betty Spooner, Steven C. Ryu, Princess C. Elhosary, Jillian M. Richardson, Danielle Tierney, Christopher A. Robinson, Rajni Chibbar, Dana Diudea, Rebecca Folkerth, Sheldon Wiebe, A. James Barkovich, Ganeshwaran H. Mochida, James Irvine, Edmond G. Lemire, Patricia Blakley, Christopher A. Walsh

**Affiliations:** 1 Division of Genetics and Genomics, Manton Center for Orphan Disease, and Howard Hughes Medical Institute, Boston Children's Hospital, Boston, Massachusetts 02115, USA;; 2 Departments of Neurology and Pediatrics, Harvard Medical School, Boston, Massachusetts 02115, USA;; 3 Broad Institute of MIT and Harvard, Cambridge, Massachusetts 02142, USA;; 4 Department of Obstetrics, Gynecology and Reproductive Biology, Brigham and Women's Hospital and Harvard Medical School, Boston, Massachusetts 02115, USA;; 5 Department of Pathology, Brigham and Women's Hospital and Harvard Medical School, Boston, Massachusetts 02115, USA;; 6 Laboratory of Molecular Medicine, Partners Personalized Medicine, Cambridge, Massachusetts 02139, USA;; 7 Institute of Child Health, University College London, London WC1N 1EH, United Kingdom;; 8 Wellcome Trust Sanger Institute, Cambridge CB10 1SA, United Kingdom;; 9 Division of Molecular Genome Analysis, German Cancer Research Center (DKFZ), 69120 Heidelberg, Germany;; 10 Department of Obstetrics and Gynecology, University of Saskatchewan College of Medicine, Saskatoon, Saskatchewan S7N 5E5, Canada;; 11 Northern Medical Services, University of Saskatchewan College of Medicine, Saskatoon, Saskatchewan S7K 0L4, Canada;; 12 Department of Pathology, Royal University Hospital, University of Saskatchewan, Saskatoon, Saskatchewan S7N 0W8, Canada;; 13 Department of Medical Imaging, Royal University Hospital, University of Saskatchewan, Saskatoon, Saskatchewan S7N 0W8, Canada;; 14 Department of Radiology, University of California San Francisco, San Francisco, California 94143, USA;; 15 Pediatric Neurology Unit, Department of Neurology, Massachusetts General Hospital, Boston, Massachusetts 02114, USA;; 16 Population Health Unit, Mamawetan Churchill River and Keewatin-Yatthé Health Regions, and Athabasca Health Authority, La Ronge, Saskatchewan S0J 1L0, Canada;; 17 Department of Pediatrics, Royal University Hospital, University of Saskatchewan, Saskatoon, Saskatchewan S7N 0W8, Canada

## Abstract

While next-generation sequencing has accelerated the discovery of human disease genes, progress has been largely limited to the “low hanging fruit” of mutations with obvious exonic coding or canonical splice site impact. In contrast, the lack of high-throughput, unbiased approaches for functional assessment of most noncoding variants has bottlenecked gene discovery. We report the integration of transcriptome sequencing (RNA-seq), which surveys all mRNAs to reveal functional impacts of variants at the transcription level, into the gene discovery framework for a unique human disease, microcephaly-micromelia syndrome (MMS). MMS is an autosomal recessive condition described thus far in only a single First Nations population and causes intrauterine growth restriction, severe microcephaly, craniofacial anomalies, skeletal dysplasia, and neonatal lethality. Linkage analysis of affected families, including a very large pedigree, identified a single locus on Chromosome 21 linked to the disease (LOD > 9). Comprehensive genome sequencing did not reveal any pathogenic coding or canonical splicing mutations within the linkage region but identified several nonconserved noncoding variants. RNA-seq analysis detected aberrant splicing in *DONSON* due to one of these noncoding variants, showing a causative role for *DONSON* disruption in MMS. We show that *DONSON* is expressed in progenitor cells of embryonic human brain and other proliferating tissues, is co-expressed with components of the DNA replication machinery, and that *Donson* is essential for early embryonic development in mice as well, suggesting an essential conserved role for DONSON in the cell cycle. Our results demonstrate the utility of integrating transcriptomics into the study of human genetic disease when DNA sequencing alone is not sufficient to reveal the underlying pathogenic mutation.

Noncoding mutations, which affect gene expression, regulation, or splicing, are estimated to cause ∼15%–30% of human Mendelian disease (Cartegni et al. 2002; Faustino and Cooper 2003; Lim et al. 2011; Lewandowska 2013). This could, in fact, be an underestimate since most genetic studies focus on coding regions (the exome) and immediately adjacent intronic splice sites whose effects are simpler to predict in silico using the amino acid code and basic splicing consensus sequences (Cartegni et al. 2002; Faustino and Cooper 2003; Pagani and Baralle 2004; Lopez-Bigas et al. 2005; Cooper and Shendure 2011; Lim et al. 2011; Lewandowska 2013). In contrast, noncoding variants are harder to interpret due to the lack of a functional understanding of most noncoding elements in the genome (Pagani and Baralle 2004; MacArthur et al. 2014). Therefore, even though most of the genome and most variants identified by whole-genome sequencing are noncoding, noncoding variants causing Mendelian disease are rarely identified before the affected gene has already been implicated by coding mutations. Although most gene regulation occurs outside of the exome—in noncoding regions such as promoters, enhancers, untranslated regions (UTRs), introns, and intergenic regions—if a genetic disease is caused by a noncoding mutation in a novel gene, it may not be possible to identify it with DNA sequencing alone among the large background of other noncoding variants. Furthermore, mutations in coding regions may also affect gene expression and splicing in addition to the protein sequence (Cartegni et al. 2002; Lopez-Bigas et al. 2005; Lewandowska 2013), effects that cannot be detected by DNA sequencing alone. As a result, even while high-throughput DNA sequencing technologies have led to remarkable progress in identifying mutations causing genetic diseases, important genetic disorders remain unsolved in which this approach fails, presumably because the pathogenic mutation is noncoding or is a coding region mutation affecting transcription.

Transcriptome sequencing (RNA-seq) has been key to revealing the complexity of gene regulation across cell types and states by its ability to profile transcript levels as well as alternative splicing patterns genome-wide (Pan et al. 2008; Wang et al. 2009; de Klerk and ‘t Hoen 2015), processes largely dictated by the noncoding genome. Nevertheless, transcriptome sequencing has not been an integral part of the gene discovery framework of human Mendelian disease studies. This might be because transcriptome sequencing ideally requires RNA from the diseased tissues of affected individuals, which is not always available, and entails the added cost and complexity of RNA sequencing and analysis relative to DNA sequencing alone.

We aimed to test whether RNA-seq could discover, in one experiment, a pathogenic noncoding mutation or transcription-altering exonic mutation causing human disease, without needing to test a large or unfeasible number of possible splicing or gene regulation defects by traditional molecular biology methods. We tested this approach by applying it to microcephaly-micromelia syndrome (MMS), a condition for which we had highly significant statistical linkage to a genetic locus but for which no obvious pathogenic coding or splicing mutation had been found by DNA sequencing.

MMS was first described in 1980 by Ives and Houston (Ives and Houston 1980) in a First Nations population in northern Saskatchewan in Canada. The syndrome's main clinical features are intrauterine growth restriction (IUGR), severe microcephaly, craniofacial dysmorphism, and marked limb malformations. Since the 1950s, an average of two pregnancies or births per year have been diagnosed with MMS in this population and, with the exception of two known children, all were stillborn or died within the first week of life, mostly within 24 h of birth. No cases of MMS have been identified outside of this First Nations population in Saskatchewan. While the clinical phenotype of MMS is distinctive, the constellation of IUGR, microcephaly, and limb anomalies places MMS in the broad category of microcephalic primordial dwarfism (MPD) syndromes, which have been associated with defects in genes involved in genome replication, the DNA damage response, and centrosome function (Klingseisen and Jackson 2011).

In this study, we sought to identify the genetic cause of MMS using a combined RNA-seq plus genome sequencing approach. We took this approach as a proof of principle to gauge the advantages and feasibility of integrating transcriptomics with genomics to reveal pathogenic noncoding mutations in unsolved human Mendelian diseases.

## Results

### Clinical features of microcephaly-micromelia syndrome

**Figure 1.**
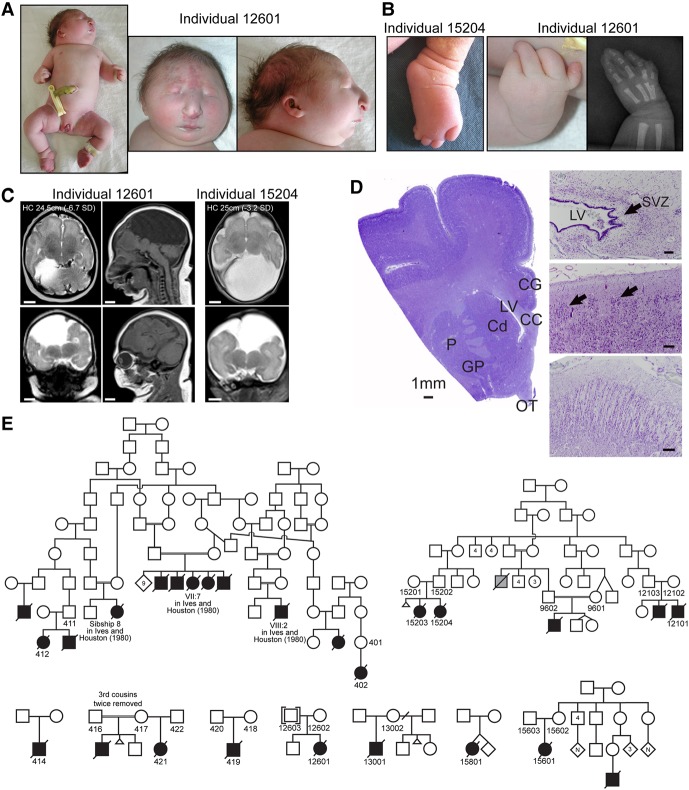
Microcephaly-micromelia syndrome phenotype and pedigrees. (*A*) Photographs of an affected individual (12601) illustrating the severe microcephaly, facial dysmorphisms, and limb anomalies characteristic of microcephaly-micromelia syndrome. (*B*) Photograph of a foot (individual 15204) and photograph and X-ray of a hand (individual 12601) showing both pre-axial (malformed toe and absent thumb) and post-axial (underdeveloped fifth metatarsal bone, and hypoplastic fifth digit arising from bifid fourth metacarpal bone) abnormalities. (*C*) Brain MRIs of two affected individuals showing the common structural brain abnormalities of MMS: profound microcephaly, simplified gyral pattern, markedly diminished white matter volume and myelination, hypoplastic or absent corpus callosum, aqueductal stenosis, and a small pons. Note the large dorsal interhemispheric cysts in both individuals, which was present in nearly every affected individual examined by MRI or autopsy to date. Cortical thickness is grossly normal and the cerebellar hemispheres are relatively large compared to the rest of the brain. Head circumferences (HC) and *z*-scores (number of standard deviations [SD] from the mean of newborns of the same gestational age at birth) are shown. MRI sequences were as follows: 12601: axial T2 (*top left*), mid-sagittal T1 (*top right*), left-sagittal T1 (*bottom right*), coronal T2-FIESTA (*bottom left*); 15204: axial T2 (*top*), coronal T2-HASTE (*bottom*). White scale bars = 1 cm. (*D*) Brain histology of MMS cases. (*Left*) Low-power Nissl-stained brain section of a child who died at 3 mo of age showing simplified gyral pattern and reduced white matter (CG) cingulate gyrus, (CC) corpus callosum, (LV) lateral ventricle, (Cd) caudate, (P) putamen, (GP) globus pallidus, (OT) optic tract). (*Top right*) Cresyl violet-stained brain section of a 35-wk-gestation newborn at the angle of the lateral ventricle (LV) showing decreased cells in the subventricular zone (SVZ; arrow). Bar = 100 μm. (*Middle right*) Hematoxylin- and eosin-stained section of the cerebral cortex of a 41-wk-gestation newborn demonstrating disorganized clusters of neurons (arrows) separated by cell-free zones in superficial layers. Bar = 100 μm. (*Bottom right*) Cresyl violet-stained section from a full-term newborn cerebral cortex demonstrating the persistence of radial columns of neurons separated by cell-sparse regions. Bar = 500 μm. (*E*) Pedigrees of the families with MMS profiled in this study. Individual IDs are labeled for individuals whose samples were profiled. The pedigree at the *top left* can be linked via individuals VII:7 and VIII:2 to the larger pedigree in the original description of the syndrome by Ives and Houston (Ives and Houston 1980). Gray symbol (*top right* pedigree) represents a child that died in infancy with limb anomalies, but the specific diagnosis of MMS was not confirmed. Deceased status is indicated with crossed-out symbols for affected individuals only and not for unaffected individuals. For simplicity, not all individuals of the pedigrees are illustrated. See Supplemental Data 1 for a list of all case samples in this study.

MMS (MIM 251230) is characterized by IUGR, marked microcephaly with distinctive craniofacial features, limb malformations, and nearly uniform perinatal lethality due to respiratory failure. The growth restriction and microcephaly are severe, with term (≥38-wk gestation) birth weights between 0.8–1.6 kg (average 1.2 kg; average *z*-score −6.5; *n* = 15), average head circumferences of 24 cm (*z*-score −7.4; *n* = 7), and average lengths of 34 cm (*z*-score −7.4; *n* = 12). Affected individuals have a characteristic facial appearance with a broad and beaked nose, short palpebral fissures, microstomia, micrognathia, low-set ears, and a short neck (Fig. 1A). Both upper and lower limbs are malformed, with upper limbs more severely affected—forearms are short, with frequent absence or significant underdevelopment of the radius and/or ulna and often humeroradial synostosis (Fig. 1A). Nearly all individuals have bilateral oligodactyly with absent thumbs, and most have absent or poorly developed fifth fingers that sometimes arise from a bifid metacarpal bone (Fig. 1B). Lower limbs are sometimes shortened with an underdeveloped fibula, feet are often clubbed with variable toe syndactyly, the great toes can be short and/or proximally placed, and in some cases, the fourth and fifth metatarsal bones and toes are underdeveloped or absent (Fig. 1B). Additionally, many affected individuals have complete craniosynostosis and other skeletal anomalies such as absence of one or two pairs of ribs.

Brain MRIs of individuals with MMS show several characteristic anomalies, including profound microcephaly with only primary sulci and gyri, diminished white matter, a hypoplastic or absent corpus callosum, aqueductal stenosis, and a large interhemispheric cyst; other gross brain structures are present, and the cerebellum is relatively preserved (Fig. 1C). Histological analysis of the cerebral cortex shows a simplified gyral pattern, reduced white matter, decreased cells in the subventricular zone, and a disorganized distribution of cells with clusters of neurons and vertically oriented columns of neurons separated by cell-sparse zones (Fig. 1D).

The lungs of individuals with MMS are severely hypoplastic with anomalous lobation. As a result, nearly all cases identified to date were either stillborn or died within the first week of life due to respiratory failure; the only known exceptions are two affected children who died at 3 mo and 2 yr of age from respiratory complications. Additionally, cleft palate and cardiac, gastrointestinal, and genitourinary defects are observed in some cases. While IUGR, microcephaly, and dwarfism are also seen in MPD syndromes (Klingseisen and Jackson 2011), the combination of the characteristic craniofacial anomalies, limb malformations, and neonatal lethality is distinct and diagnostic for MMS.

### Microcephaly-micromelia syndrome is linked to a locus on Chromosome 21q

**Figure 2.**
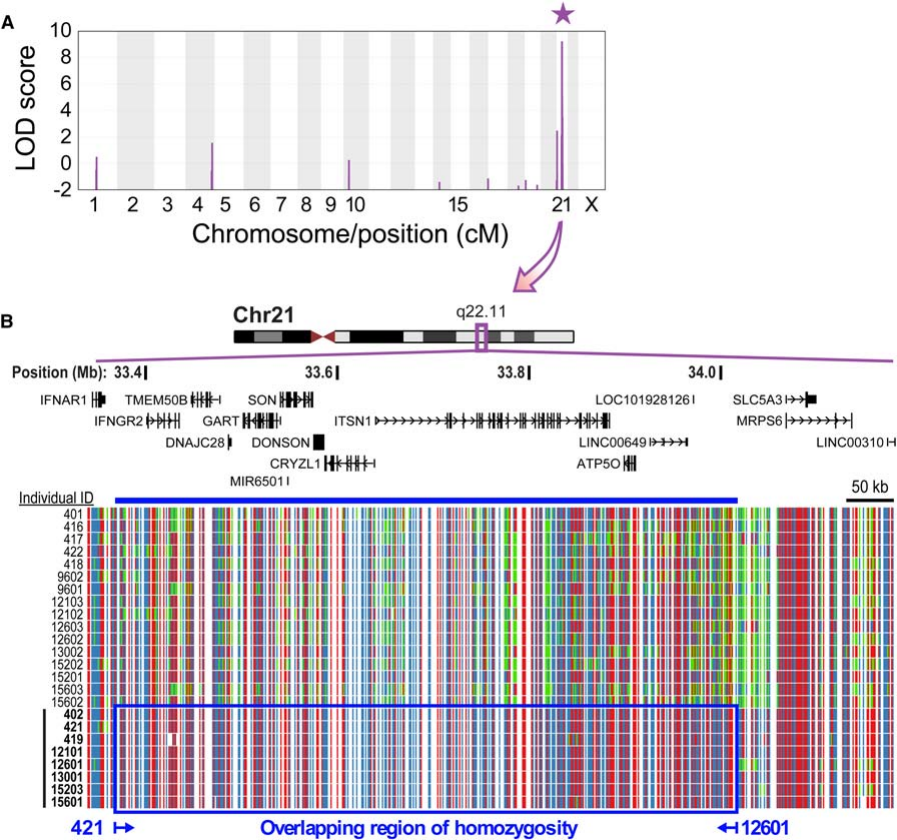
Linkage and homozygosity analysis identifies a locus on Chromosome 21q22.11. (*A*) Linkage analysis using SNP-microarray genotypes of affected individuals and unaffected parents (see Supplemental Data 1 for list of genotyped individuals) identified a locus associated with the disease at Chromosome 21q22.11 with maximum LOD score of 9.2 (purple star; interval: Chr 21: 33,344,469–34,196,070; GRCh38/hg38). (*B*) SNP-microarray genotypes in the interval defined by linkage analysis (Fig. 2A). Each line represents an individual (unaffected parents on *top* and affected individuals labeled in bold on *bottom*). Each column in the SNP ideogram represents a SNP, with homozygous alleles in red or blue and heterozygous alleles in green. Affected individuals 421 and 12601 define a minimal region of overlapping homozygosity (ROH) at 21q22.11 (blue box and line; Chr 21: 33,364,965–34,029,433; GRCh38/hg38). RefSeq gene annotations are shown above. Low quality SNP calls are omitted. Note: samples 418 and 419 are shown here but these were not used for linkage analysis since they did not pass quality control filters (see Supplemental Methods).

Most individuals with MMS in this study belong to a large and consanguineous pedigree of First Nations origin in Saskatchewan, showing autosomal recessive inheritance (Fig. 1E). Other cases belong to the same population and are known to be descendants of the founders of this pedigree, though their exact relationships are unknown (Fig. 1E; see Supplemental Data 1 for a list of all profiled individuals). High-density genome-wide single nucleotide polymorphism (SNP)-microarray genotyping of seven affected individuals and 15 unaffected parents followed by linkage analysis under a recessive inheritance model identified an 852-kb (1.2 cM) locus on Chromosome 21q22.11 definitively associated with the disease with a maximum combined logarithm of odds (LOD) score of 9.2 (Fig. 2A). Homozygosity and haplotype analysis using SNP-microarray genotypes of affected cases further narrowed this region to a 664-kb minimal overlapping region of homozygosity (ROH) (rs9978569 to rs4443074; Chr 21: 33,364,965–34,029,433; GRCh38/hg38) (Fig. 2B).

### Multimodal genome sequencing of the disease locus fails to identify plausible exonic or canonical splice mutations

We sequenced three affected individuals using three different methods: whole-exome sequencing (WES, individuals 412 and 13001), targeted-capture sequencing of the linkage region (individual 13001), and whole-genome sequencing (WGS, individual 12601). These methods covered 97.0%, 99.8%, and 97.9%, respectively, of the coding exome in the ROH with ≥10× read depth (see Supplemental Fig. 1A for coverage statistics). Targeted-capture and WGS covered 83% and 98% of the entire ROH (i.e., coding exons, introns, UTRs, and intergenic regions), respectively, with ≥10× read depth (Supplemental Fig. 1A). The variants identified by the three methods were highly concordant, though some variants were identified by only one or two of the three methods (Supplemental Fig. 1C,D; Supplemental Data 2). After filtering out common population variants, no rare coding (either synonymous or nonsynonymous) or canonical splice site mutations (i.e., within 2 bp of the intron-exon junction) were identified within the ROH by any of these sequencing approaches (Table 1; Supplemental Fig. 1B). In contrast, 18 rare intronic and 20 rare intergenic noncoding variants were identified in the ROH (Table 1; Supplemental Fig. 1B; Supplemental Data 2). Analysis of highly conserved noncoding elements in the region did not aid in further narrowing the list of noncoding variants.

**Table 1.**
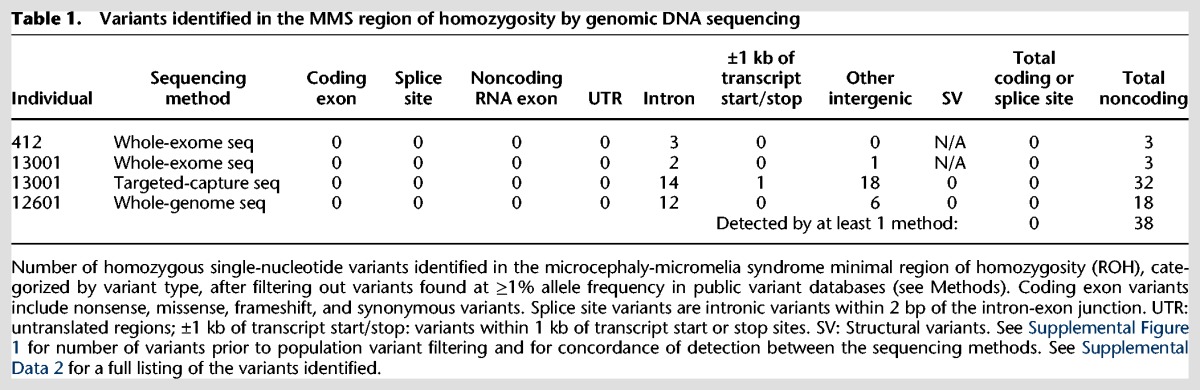
Variants identified in the MMS region of homozygosity by genomic DNA sequencing

Furthermore, copy number variant (CNV) and structural variant analyses were performed using the targeted-capture and WGS data. CNV analysis was also performed by a targeted array comparative genomic hybridization experiment. None of these methods identified a CNV or structural variant in the ROH (Table 1; Supplemental Fig. 1B).

### RNA-seq identifies a splicing defect associated with an intronic variant in *DONSON*

Since comprehensive genome-sequencing did not reveal a coding or canonical splicing mutation in the MMS ROH, we hypothesized that one of the 38 noncoding variants detected in the ROH may be the cause of the disease and that transcriptome profiling of affected samples could reveal which of these noncoding mutations causes MMS via *cis* effects on gene expression or splicing. We therefore performed RNA-seq of wild-type control (*n* = 13), heterozygous parent (*n* = 6), and homozygous MMS case samples (*n* = 11) derived from a variety of cell lines and tissues.

**Figure 3.**
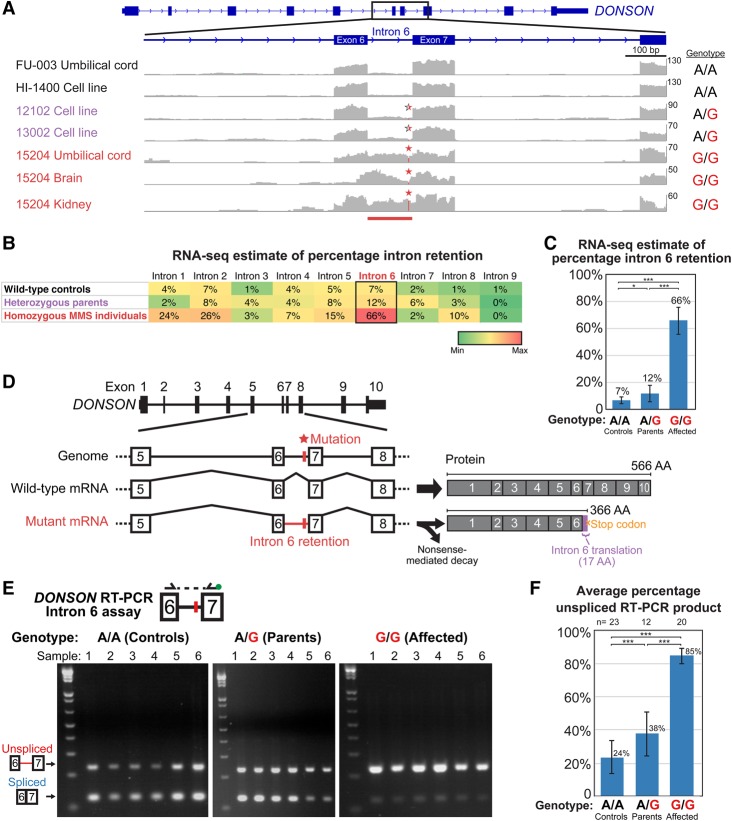
RNA-seq identifies an intron-retention splicing defect associated with an intronic variant in *DONSON*. (*A*) RNA-seq read coverage for representative samples shows aberrant retention of intron 6 of *DONSON* (red bar) in affected individuals associated with the c.1047-9A>G noncoding mutation (Chr 21: g.33582064:T>C; red asterisk). The interval shown is Chr 21: 33,580,994–33,583,594 (GRCh38/hg38), illustrated in the reverse strand direction. The genotype of each sample is shown on the *right*. Homozygous MMS, heterozygous parent, and wild-type control sample names are colored red, purple, and black, respectively. Read coverage graph *Y*-axes are scaled (numbers on right side of *Y*-axis) to show the maximum coverage of each sample in the interval. (*B*) RNA-seq quantification of intron retention for each intron of *DONSON*, based upon pooling all the RNA-seq samples of each genotype (see Supplemental Methods for details). Cells are shaded green to red according to the percentile between the minimum and maximum values in the table. The aberrant retention of intron 6 is highlighted. The table also shows the mild increase in intron 6 retention in heterozygous parents relative to wild-type controls and the baseline low-level retention of intron 6 in controls relative to other introns. MMS individuals also showed a trend of increased retention of other introns upstream of intron 6, suggesting that impaired splicing of intron 6 might affect splicing of other introns; however, the mechanism by which this would occur is unclear. (*C*) RNA-seq quantification of intron 6 retention calculated as in Figure 3B. Error bars are 95% confidence intervals (see Supplemental Methods). All group comparisons were significant: controls versus parents: *P* = 0.03; controls versus affected: *P* < 10^−15^; parents versus affected: *P* < 10^−15^ (Fisher's exact test with Holm multiple comparisons adjustment). (*D*) Schematic of the intron retention splicing defect caused by the c.1047-9A>G (Chr 21: 33582064 T>C) mutation in intron 6 in microcephaly-micromelia syndrome. Retention of intron 6 would lead to either nonsense-mediated decay of the transcript due to the stop codon within intron 6 or to a truncated protein. On the right are the predicted wild-type and truncated mutant proteins and their amino acid (AA) lengths. Translation of the first part of the aberrantly retained intron 6 creates 17 amino acids followed by a premature stop codon. (*E*) RT-PCR spanning from exon 6 to exon 7 of *DONSON* (*top* schematic) in various tissues confirms increased retention of intron 6 in MMS samples, which are homozygous for the Chr 21: 33582064 T>C mutation, compared to heterozygous parents and wild-type controls (unspliced transcript with intron 6: 230 bp; spliced transcript: 121 bp). Shown here are six representative samples for each genotype. (Note that the variant is A>G in the *DONSON* transcript strand and T>C in the genomic plus strand). See Supplemental Figure 2A for RT-PCR gel images of all assayed samples. The exon 7 PCR primer contains a FAM fluorescent label (green circle) for quantification of PCR products (Fig. 3E). Wild-type (T/T) samples: 1- FU-009 umbilical cord; 2- FU-006 umbilical cord; 3- FU-004 umbilical cord; 4- fetal liver; 5- fetal brain; 6- cerebellum. Heterozygous (T/C) parent samples: 1- 15603 cell line; 2- 15602 cell line; 3- 15202 cell line; 4- 15201 cell line; 5- 15202 blood sample a; 6- 15202 blood sample b. Homozygous (C/C) MMS samples: 1- 15204 brain (RNAlater); 2- 15204 brain (fresh-frozen sample a); 3- 15204 brain (fresh-frozen sample b); 4- 15204 heart (RNAlater); 5- 15204 heart (fresh-frozen); 6- 15204 kidney (RNAlater). (*F*) Quantification of the RT-PCR intron 6 retention assay products. RT-PCR was performed with the PCR primer for exon 7 containing a FAM-fluorescent label allowing quantification of the RT-PCR products with a capillary electrophoresis DNA analyzer (see Methods). Percentage unspliced RT-PCR product was calculated as [Area of unspliced band]/[Area of unspliced band + Area of spliced band], and averaged across all samples of each genotype (number of samples in each group is shown on *top*). Groups were significantly different from each other (Controls versus Parents, *P* = 0.005; Controls vs. Affected, *P* < 10^−22^; Parents vs. Affected, *P* < 10^−7^; two-tailed unpaired *t*-test). Importantly, note that this measurement can be used to evaluate relative splicing differences between genotypes but is not an absolute measurement of splicing, since the PCR amplification efficiencies of the unspliced and spliced products differ. See Supplemental Figure 2A for percentage unspliced RT-PCR product of all assayed samples and Supplemental Figure 2B for percentage unspliced RT-PCR product summarized by tissue type for brain, umbilical cord, and cell lines/blood leukocytes.

RNA-seq analyses of gene expression and splicing in the 30 samples from affected and unaffected individuals identified only one abnormality within the ROH in MMS samples: significantly increased retention of intron 6 of *DONSON* in MMS samples relative to heterozygous parent and wild-type control samples (Fig. 3A). Importantly, intron 6 of *DONSON* contained one of the 38 noncoding variants identified previously by genome sequencing, an A to G transition (in the transcript sense strand) located 9 bp upstream of the intron 6–exon 7 junction (*DONSON* [NM_017613.3]:c.1047-9A>G; Chr 21[NC_000021.8]:g.33582064T>C [GRCh38;hg38]) (Supplemental Data 2). The only two other variants in *DONSON* were located distantly in other introns, 1.2 kb (intron 5) and 2.4 kb (intron 4) away (Supplemental Data 2). Furthermore, MMS samples showed no evidence of any novel *DONSON* isoforms, such as might arise from activation of cryptic 5′ splice donor or 3′ splice acceptor sites within intron 6. Retention of intron 6 creates a premature stop codon after 52 bp of the 109-bp intron, which is predicted to lead to nonsense-mediated decay (NMD) of the mutant transcript (Wong et al. 2016) or to a truncated protein (366 amino acids versus wild-type 566 amino acids) that ends with 17 aberrant amino acids due to translation of the first half of intron 6 (Fig. 3D). Overall, *DONSON* expression levels in RNA-seq showed a trend toward lower expression in heterozygous parents and homozygous MMS cases, but these differences were not statistically significant (controls: 5.2 ± 2.8 fragments per kilobase per million mapped reads [FPKM mean ± std dev], parents: 3.9 ± 2.4 FPKM, MMS cases: 3.4 ± 1.7 FPKM; *P*-value >0.05 for all comparisons). However, a more sensitive TaqMan qPCR assay confirmed significantly decreased *DONSON* transcript levels in the presence of the MMS variant (56% of wild-type levels in heterozygous cell lines; 95% confidence interval: 44%–72%) (Supplemental Fig. 2C), consistent with NMD of the mutant transcript. Altogether, these results suggest that MMS is caused by the c.1047-9A>G noncoding variant in *DONSON* via an intron retention mechanism.

Genotyping of the intron 6 noncoding variant across all available samples from the extended MMS pedigrees confirmed it was homozygous in all affected individuals and heterozygous in all parents (Supplemental Data 1). The variant was absent from the NHLBI Exome Sequencing Project (6503 individuals), the 1000 Genomes Project, a set of 69 Complete Genomics control genomes, and 1464 unrelated exomes previously sequenced by our laboratory. The variant was found in the heterozygous state in one of 736 additional neurologically normal control samples that we genotyped and in six of 121,390 chromosomes in the Exome Aggregation Consoritum (ExAC) database, all in the heterozygous state in European individuals (Supplemental Data 2). The locus of this intronic variant is captured well by exome sequencing due to its relative proximity to an intron-exon junction (e.g., the locus was called in 121,390 of 121,412 chromosomes in ExAC), so its low frequency in control exomes was not due to inefficient capture. The extremely low allele frequency of the variant across these studies (5 × 10^−5^), along with the unique hypomorphic nature of the allele and the essential requirement for *DONSON* in body development (see below analyses), is consistent with the extreme rarity of the microcephaly-micromelia syndrome.

In order to independently confirm and quantify the splice defect identified by RNA-seq, we designed a reverse transcriptase-polymerase chain reaction (RT-PCR) assay for intron 6 retention using primers flanking intron 6, from exon 6 to exon 7. The assay confirmed the intron 6 retention defect in all MMS samples and showed a mild but detectable increase in intron retention in heterozygous parents relative to control samples (Fig. 3E; Supplemental Fig. 2A). Interestingly, the assay also showed that splicing of intron 6 is not perfectly efficient in control samples, with low and variable levels of intron 6 retention in normal tissues, though significantly less than in the MMS samples (Fig. 3E; Supplemental Fig. 2A). The RT-PCR assay employed a fluorescent primer (Fig. 3E schematic), allowing quantification of the two RT-PCR products (spliced product and intron 6 retention product). The intron 6 retention amplicon was, on average, 85 ± 5% (standard deviation) of the total RT-PCR product in homozygous MMS samples, 38 ± 13% in heterozygous parent samples, and 24 ± 10% in wild-type control samples (Fig. 3F). All differences in intron 6 splicing between the groups of samples were statistically significant (controls vs. parents, *P* = 0.005; controls vs. affected, *P* < 10^−20^; parents vs. affected, *P* < 10^−5^; two-tailed unpaired *t*-test with Holm multiple comparisons adjustment) (Fig. 3F). These RT-PCR results confirm a significant intron 6 retention splice defect of *DONSON* in MMS samples, a small but detectable increase in intron 6 retention in heterozygous parents, and that intron 6 splicing is not perfectly efficient in normal tissues.

The above RT-PCR assay allows robust comparisons of the relative efficiency of intron 6 splicing between samples; however, because the RT-PCR amplification efficiency of the spliced and unspliced products may differ due to their different sizes, the assay might not be a fully accurate measure of absolute splicing efficiency. We therefore also estimated intron 6 retention using the RNA-seq data by counting the number of intron–exon junction spanning reads (reflecting intron retention) versus exon 6–exon 7 splice reads (reflecting intron splicing). In MMS samples, 66% of these reads at the intron 6 locus were intron–exon junction spanning reads that arose from *DONSON* transcripts retaining intron 6, versus 12% in heterozygous parents and 7% in controls (Fig. 3A–C). The differences between each category of samples were statistically significant (controls versus parents: *P* = 0.03; controls versus affected: *P* < 10^−15^; parents versus affected: *P* < 10^−15^; Fisher's exact test with Holm multiple comparisons adjustment). Furthermore, in heterozygous parent samples, more intron 6-retaining transcripts were derived from the mutant allele than the wild-type allele: 69% of RNA-seq reads at the intron 6 mutation locus (pooled from all heterozygous samples) contained the mutation (*P* = 0.02; binomial test versus the expected 50% if there were no association between the intron retention and the mutant allele). The above RNA-seq results again confirm that (1) individuals with MMS have a hypomorphic *DONSON* allele in which most transcripts retain intron 6, while a small fraction of transcripts are correctly spliced, (2) there is a small increase in intron 6 retention in heterozygous parents relative to controls, (3) the intron 6 variant is associated in *cis* with intron 6 retention, and (4) there is baseline low-level retention of intron 6 in wild-type tissues.

Notably, plotting of intron retention estimated by RNA-seq across all introns of *DONSON* and all sample types showed not only the specific intron 6 retention defect, but also that in wild-type control tissues, intron 6 is one of the gene's least efficiently spliced introns (Fig. 3B). Therefore, the low level of intron 6 retention seen in control samples by RNA-seq and RT-PCR is not due to overall splicing inefficiency in the assayed samples or global capture of unspliced transcripts during RNA-seq sample preparation, but rather a specific feature of intron 6. The baseline decreased efficiency of intron 6 splicing compared to other introns might explain the intron's susceptibility to the MMS c.1047-9A>G intronic mutation.

### *DONSON* is essential for early embryonic development and is associated with components of the DNA replication machinery

In order to determine how the MMS phenotype relates to *DONSON* function during development, we assessed *DONSON* expression in human and mouse embryos by in situ hybridization. In both human and mouse, we found that *DONSON* is expressed in multiple organs of the developing embryo, including the brain, heart, lungs, gastrointestinal tract, limbs, and kidneys (Supplemental Figs. 3A–C), which grossly corresponds to the organs affected in MMS.

**Figure 4.**
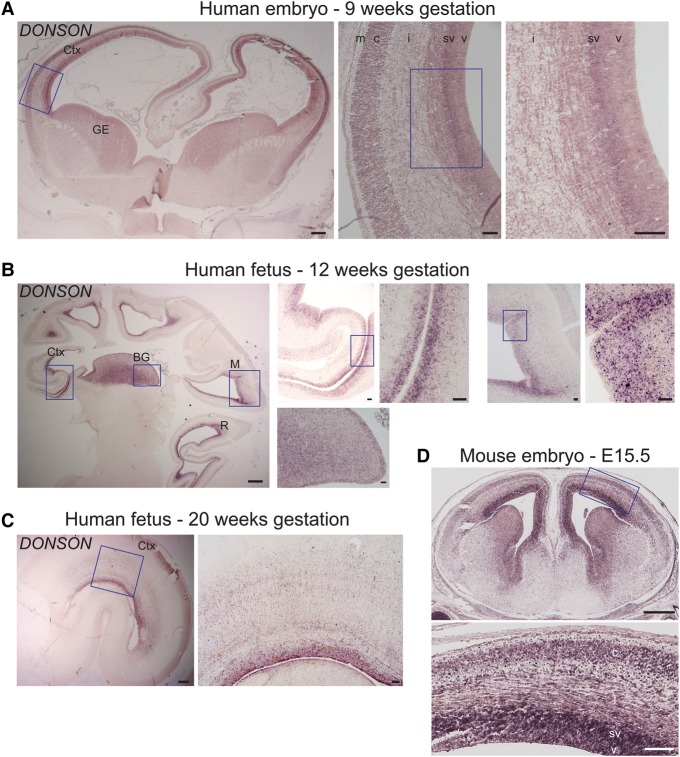
*DONSON* expression in human and mouse brain development. (*A*) *DONSON* expression by in situ hybridization in a coronal section of a 9-wk-gestation human fetal brain. Expression is prominent in the neocortex subventricular zone, which contains progenitor cells, and in the cortical plate, where newly born neurons reside. Expression is also seen in the ganglionic eminences, which give rise to the basal ganglia and interneurons that migrate into the neocortex. Scale bar for main image (*left*) is 500 µm; scale bars for other images are 100 µm. Hybridization was performed with antisense probe 1 (see Supplemental Methods). (Ctx) Neocortex, (GE) ganglionic eminence, (v) ventricular zone, (sv) subventricular zone, (i) intermediate zone, (c) cortical plate, (m) marginal zone. (*B*) *DONSON* expression by in situ hybridization in a sagittal section of a 12-wk-gestation human fetal brain. Expression is prominent in the basal ganglia (BG) and the ventricular and subventricular zones and cortical plate of the neocortex (Ctx), mesencephalon (M, midbrain), and rhombencephalon (R, hindbrain). Scale bar for main image (*left*) is 1000 µm; scale bars for other images are 100 µm. Hybridization was performed with antisense probe 1. (*C*) *DONSON* expression by in situ hybridization in a coronal section of a hemisphere of a 20-wk-gestation human fetal brain. Expression is evident in the ventricular and subventricular zones, intermediate zone, and cortical plate of the neocortex (Ctx). Scale bar for main image (*left*) is 1000 µm; scale bar for other image is 100 µm. Hybridization was performed with antisense probe 2. (*D*) *DONSON* expression by in situ hybridization in a coronal section of an E15.5 mouse brain. Expression is evident in the ventricular (v) and subventricular zones (sv), intermediate zone (i), and cortical plate (c) of the neocortex. Scale bar for *top* and *bottom* images are 500 and 100 µm, respectively. Hybridization was performed with human antisense probe 3. For all above tissue sections, negligible signal was observed with sense sequence probes in adjacent sections (Supplemental Fig. 3D), confirming specificity of the antisense probe staining.

Because MMS has a severe microcephaly phenotype, we assessed *DONSON* expression in greater detail in human and mouse embryonic and fetal brains. These in situ hybridization studies found that *DONSON* is highly enriched in the ventricular and subventricular zones of the neocortex, which contain proliferating progenitor cells, as well as in the cortical plate containing newborn neurons, and the ganglionic eminences (Fig. 4A–D). This pattern of expression is consistent with the observed simplified gyral pattern, reduced white matter, and disorganized cortical columns seen in histology of MMS brains (Fig. 1D). We also investigated *DONSON* expression in a prior transcriptomic study of laser-microdissected regions of the developing human brain (Miller et al. 2014) and found a similar pattern of expression: in 15- and 16-wk-post-conception (wpc) human fetal brains, *DONSON* is highly expressed in the ventricular and subventricular zones, respectively; it is highly expressed in the ganglionic eminence of 15-wpc brain and subplate of 16-wpc brain, and in 21-wpc neocortex, *DONSON* expression is highest in the cortical plate (Supplemental Fig. 4A). Loss of *DONSON* function in early neocortical progenitor cells is consistent with the profound microcephaly and brain malformations of MMS. Furthermore, assays of *DONSON* expression across a broad range of prenatal and adult time points as part of the Human Brain Transcriptome project (Kang et al. 2011) show that *DONSON* expression is higher in prenatal brain relative to adult brain across all profiled brain regions (Supplemental Fig. 4B), supporting an important role for DONSON specifically during brain development.

To confirm the essential role of DONSON in prenatal development, we also analyzed the phenotype of *Donson* knockout mice, which were created as part of the International Knockout Mouse Consortium. Matings between mice heterozygous for a *Donson* loss-of-function allele did not yield any homozygous pups, and genotyping of E9.5, E12.5, and E14.5 embryos did not detect any homozygous embryos (Table 2), indicating that complete loss of DONSON function is lethal during early murine embryonic development. Heterozygous knockout mice were phenotyped on >150 anatomic and laboratory measures (including brain weight) via the standardized International Mouse Phenotyping Consortium protocol (Supplemental Methods; White et al. 2013), with no major discernible abnormalities detected, though it is possible that subtle brain phenotypes could be revealed by additional detailed neuroanatomic studies. The essential role of DONSON in organismal development is also supported by phylogenetic analyses—DONSON is conserved across all multicellular eukaryotes, with orthologs found in mammals, birds, reptiles, insects, roundworms, plants, and fungi (Supplemental Fig. 5B; Bandura et al. 2005).

**Table 2.**
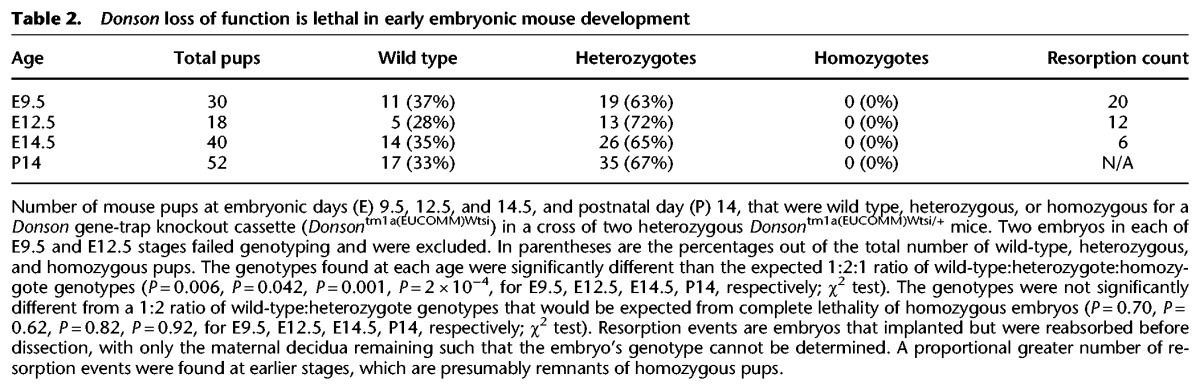
*Donson* loss of function is lethal in early embryonic mouse development

We utilized public proteomic and gene expression databases to identify complexes and proteins with which DONSON may function in the cell cycle. The *Drosophila* Protein Interaction Map project, which used tagged proteins to profile the *Drosophila* proteome, found that Humpty Dumpty, DONSON's ortholog in fly, is the highest-ranked interactor of Asf1 (Guruharsha et al. 2011; Chatr-Aryamontri et al. 2015). Asf1 is a histone chaperone that coordinates nucleosome assembly on newly replicated DNA with chromatin unwinding by the MCM2-7 prereplicative and replication helicase complexes. This points to a role for DONSON in the genome replication machinery.

Next, we mined the COXPRESdb database (Okamura et al. 2015), which collates thousands of microarray and RNA-seq samples from various species to create robust lists of co-expressed genes, and found further independent validation of an association between DONSON and components of the DNA replication and replication fork machinery. In *Drosophila*, more than half of the top 20 genes most highly co-expressed with *humpty dumpty* have known roles in the DNA replication complexes, including (1) DNA polymerase subunits involved in initiation of replication: *DNApol-alpha73* [*POLA2*] and *DNApol-alpha60* [*PRIM2*] (human homologs in brackets), (2) DNA polymerase processivity factors: *RfC38* [*RFC3*], *CG8142* [*RFC4*], and *CG11788* [*DSCC1*] that help load the PCNA sliding clamp, which mediates polymerase processivity, onto primed DNA (Bermudez et al. 2003; Bowman et al. 2004), (3) DNA ligase 1 (*DNA-ligI* [*LIG1*]), which joins Okazaki fragments during DNA replication and is also associated with PCNA, (4) components of the DNA replication pre-initiation and replication fork complexes: *CG3430* [*MCMBP*], *Mcm3* [*MCM3*], *Cdc45* [*CDC45*], (5) *Orc2* [*ORC2*], a component of the origin recognition complex, and (6) regulators of the cell cycle: *Lethal-(2)-denticleless* [*DTL*]—which codes for a ubiquitin ligase targeting key cell cycle regulators and associated with PCNA and the DNA replication licensing factor CDT1 (Higa et al. 2006), and *Cortex*—a member of the Cdc20/fizzy family of cell cycle regulators (Nadeau et al. 2016).

In the human COXPRESdb data, the top genes most highly co-expressed with *DONSON* were again strikingly enriched for genes coding for components of the DNA replication machinery, including (1) the *ORC1* origin recognition complex subunit 1, (2) *CDC6*, which together with CDT1 loads the MCM2-7 replication helicase onto replication origins, (3) *GINS1*, a component of the active CMG (CDC45/MCM2-7/GINS) replication helicase, (4) *MCM4* and *MCM6*, components of the MCM2-7 helicase, (5) *POLE2*, a subunit of DNA polymerase-epsilon, (6) *MCM10*, an essential component of the replication fork that mediates the association of MCM2-7 and DNA polymerase-alpha with replication origins (Homesley et al. 2000; Ricke and Bielinsky 2004), (7) *CHAF1B*, a subunit of the chromatin assembly factor that loads histones onto newly replicated DNA (Volk and Crispino 2015), and (8) cell cycle regulators: *CDK1*, *DTL*, cyclin E2, and cyclin A2 (a regulator of origin of replication firing).

A similarly remarkable enrichment of replication complex genes was seen in mouse and rat COXPRESdb data, including *Orc6*, *Cdt1*, *Cdc6*, *Mcm2*, *Mcm3*, *Mcm4*, *Pcna*, *Dscc1*, *Fen1* (the endonuclease processing Okazaki fragments), and cyclin E1—all of which were within at least one of the two species’ top 20 genes most highly co-expressed with *Donson*.

Finally, to confirm an effect of *DONSON* loss on cell cycle progression, we assayed the expression of a panel of cell cycle genes (i.e., cyclins, cyclin-dependent kinases, checkpoint regulators) by qPCR after siRNA knockdown of *DONSON* in HeLa cells. After *DONSON* knockdown, the most significantly up-regulated gene in the panel was *CDKN1A* (p21), and the most significantly down-regulated genes were cyclin D2 and cyclin E2 (Supplemental Fig. 4C). Since p21 inhibits, and cyclins D2 and E2 mediate, the G1/S phase transition (Vermeulen et al. 2003), these results are consistent with arrest prior to or slowed progression through S phase seen with *DONSON* loss in fly and HeLa cells in prior studies (Bandura et al. 2005; Fuchs et al. 2010). It also suggests that p21 up-regulation mediates the cell cycle arrest triggered by *DONSON* loss. Further evidence that *DONSON* loss impairs cell proliferation is that, despite multiple attempts, we have been unable to culture cell lines from affected MMS patients—the cells die after their derivation and cannot be expanded in vitro, while multiple cell lines from their unaffected parents grow normally.

Altogether, the above data strongly suggest that DONSON is essential for genome replication, and therefore cell proliferation and early embryonic development, via a regulative or integral function in the prereplication and/or replication fork complexes, which notably, are disrupted in other MPD syndromes.

## Discussion

Here, we present a noncoding mutation in *DONSON* as the cause of microcephaly-micromelia syndrome and link DONSON to the key complexes mediating genome replication that are disrupted in other microcephalic primordial dwarfism syndromes. Identification of this unusual hypomorphic mutation—in an essential gene whose complete loss of function we predict would otherwise be embryonic-lethal—was facilitated by a genomic plus transcriptomic integrated approach that serves as a model for discovering causes of other Mendelian diseases for which genome sequencing alone has been unsuccessful.

### A transcriptomic approach facilitates the discovery of variants causing Mendelian disease

Our study illustrates how transcriptome sequencing (RNA-seq) can provide in vivo functional evidence for rapid identification of pathogenic variants, in particular those that alter gene expression or splicing. While our study identified an unexpected noncoding mutation in a novel disease gene, several prior studies have also shown the potential of RNA-seq for discovering splicing perturbations in known human disease genes (Chandrasekharappa et al. 2013), splicing defects caused by variants in canonical (i.e., predictable) splice sites of genes not previously associated with disease (Wang et al. 2013), fusion transcripts in cancer (Pierron et al. 2012; Morin et al. 2013), and RNA-seq confirmation of known transcription-altering mutations from genetic screens in model organisms (Miller et al. 2013). These studies also identified decreased transcript levels due to nonsense-mediated decay and other unexpected splice defects such as missense and synonymous exonic mutations causing exon skipping (Chandrasekharappa et al. 2013; Miller et al. 2013). We have also previously used RNA-seq to confirm a splicing defect that we had first identified by traditional molecular biology methods in the *ZNF335* gene causing microcephaly (Yang et al. 2012). RNA-seq showed that the pathogenic missense mutation in the last base of one of the gene's exons caused retention not just of the immediately following intron, but, unexpectedly, also the preceding intron. These examples emphasize the complex and unpredictable nature of splicing mutations and the utility of RNA-seq as a high-throughput adjunct assay to DNA sequencing in assessing the transcriptional impact of both coding and noncoding variants.

RNA-seq has several additional important advantages. First, RNA-seq might allow direct identification of the pathogenic mutation and transcriptional defect without prior variant filtering or linkage analysis. While in our study of *DONSON*, the retained intron 6 is short and cannot provide such genome-wide power of detection, in some cases, RNA-seq of a single proband could be sufficient for identifying the disease-causing variant. For example, in our earlier study of *ZNF335*, the retained long intron ranked as the fourth most significant differentially transcribed intron out of >350,000 RefSeq-annotated introns, and the other top entries were false-positives due to unannotated exons (Yang et al. 2012). Situations that could feasibly provide such power for genome-wide detection would be the retention of long introns and aberrant splice donor or acceptor sites, which provide greater signal to background ratios than other defects such as exon-skipping that are more common normal alternative splice events.

Second, RNA-seq can reveal allelic association of variants to abnormal transcripts, providing additional functional evidence of causality. In heterozygous samples, biased expression of an aberrant transcript from the variant allele, as seen with the *DONSON* mutation, indicates that the variant causes the abnormal transcript in *cis*. Third, RNA-seq provides a functional read-out of the entire in vivo context of variants, which is not fully recapitulated in minigene and other reporter assays.

On the other hand, RNA-seq is limited by its dependence on whether the affected gene is normally expressed in the available tissues at sufficient levels to allow detection of abnormal transcripts. The feasibility of a transcriptomic approach therefore depends on the studied disease and the accessibility of affected tissues. One possible approach when affected tissues are not available may be to create induced pluripotent stem cells from patient samples and to differentiate them into the cell types affected by the disease, thereby allowing RNA-seq analysis of the disease-relevant mRNA species.

### Intron retention as a mechanism of genetic disease

While progress has been made in understanding the mechanisms of RNA splicing and how splicing patterns relate to DNA sequence (Barash et al. 2010; Xiong et al. 2015), much of the genomic code mediating splicing remains unknown (Lewandowska 2013; Guigo and Valcarcel 2015; Lee and Rio 2015). Intron retention in particular has been the least studied alternative splicing pattern. Recent work, however, has shown that intron retention might be a conserved and more common mode of transcript regulation than previously appreciated (Galante et al. 2004; Braunschweig et al. 2014; Boutz et al. 2015; Mudvari et al. 2015; Wong et al. 2016), with examples found in hematopoiesis (Wong et al. 2013; Edwards et al. 2016), T-cell activation (Ni et al. 2016), and neuronal development and activity (Bell et al. 2010; Yang et al. 2012; Yap et al. 2012). Inappropriate intron retention has also been identified in diverse genetic diseases, including cancer (Seifert et al. 2006; Tanackovic et al. 2011; Dvinge and Bradley 2015; Jung et al. 2015; Ortega-Recalde et al. 2015; Kallabi et al. 2016). Although there are tools that can predict the effects of sequence variants on alternative exon use or alternative 5′ donor or 3′ acceptor splice sites (Jian et al. 2014), to our knowledge, there are no computational tools to predict intron retention from sequence data. The increasingly appreciated role of intron retention in normal physiology and disease therefore supports the need for empirical methods such as transcriptomics to address this gap.

Intron retention may regulate normal gene expression or cause disease not only by introducing additional protein sequence, but also by NMD or by preventing export of the transcript from the nucleus if the retained intron introduces a premature stop codon (Ge and Porse 2014; Wong et al. 2016). The trend toward lower *DONSON* levels in MMS samples suggests that the intron retention leads to at least some NMD. Interestingly, in normal tissues, intron 6 of *DONSON* is both less efficiently spliced out compared to other introns and is variably spliced across tissues. This suggests that perhaps, more generally, inefficiently spliced introns might be more vulnerable to disruption by genetic mutation.

### A nonconserved noncoding variant causing disease

Evolutionary conservation is often used to predict the likelihood of variant pathogenicity. However, while *DONSON* is conserved across all multicellular eukaryotes (Supplemental Fig. 5A,B), the wild-type allele at the location of the MMS intronic mutation is not well conserved (Supplemental Fig. 5A). This illustrates that conservation analyses cannot be exclusively relied upon to predict the clinical significance of noncoding variants.

### *DONSON* is associated with genome replication complexes disrupted in other microcephalic primordial dwarfisms

Clinically, MMS is a microcephalic primordial dwarfism, a class of heterogeneous disorders characterized by prenatal and postnatal growth restriction along with microcephaly (Klingseisen and Jackson 2011; Khetarpal et al. 2016). MPD syndromes are classified into four types based on their clinical features and affected cellular pathways, all of which are involved in some aspect of cell cycle progression (Klingseisen and Jackson 2011; Fenwick et al. 2016; Khetarpal et al. 2016). Seckel syndrome is caused by mutations in DNA damage response signaling and centriole biogenesis factors (*ATR*, *ATRIP*, *CEP152*, *CENPJ*). Microcephalic osteodysplastic primordial dwarfism (MOPD) type I/III is caused by mutation of *RNU4ATAC*, a component of the minor spliceosome mediating U12-intron splicing in many genes including some involved in DNA replication. MOPD type II is caused by mutations in *PCNT* encoding a key centrosomal protein. Meier-Gorlin syndrome (MGS) is caused by mutations in components of the prereplication and pre-initiation DNA replication complexes (*ORC1*, *ORC4*, *ORC6*, *CDT1*, *CDC6*, *GMNN*, *CDC45*), which license replication origins and mediate loading and functioning of the replication fork helicase (Bicknell et al. 2011; Burrage et al. 2015; Fenwick et al. 2016). Although MMS is distinct from these four classic types of MPD syndromes, in this study we have presented genetic, transcriptomic, and proteomic evidence linking MMS and *DONSON* to other MPD syndromes, in particular, MGS.

Multiple independent lines of evidence strongly suggest an essential role for DONSON in the same DNA prereplication and replication fork complexes affected in MGS. First, expression of both human DONSON and its fly ortholog Humpty Dumpty (Hd) peaks in late G1 and S-phase when origins of replication complexes assemble and genome replication takes place (Whitfield et al. 2002; Bandura 2005; Bandura et al. 2005; Fuchs et al. 2010). Second, endogenous Hd localizes to the nucleus in foci that overlap, though not exclusively, with origins of replication (Bandura et al. 2005). Third, genetic loss of Hd in fly and siRNA knockdown of human *DONSON* leads to impaired genome replication, arrest prior to or slowed progression through S-phase of the cell cycle, and impaired cell proliferation (Bandura 2005; Bandura et al. 2005; Fuchs et al. 2010). Hd and DONSON depletion also lead to an increase in histone variants marking DNA double-strand breaks, which could be a consequence of stalled replication forks (Bandura et al. 2005; Fuchs et al. 2010). Notably, Hd-null fly mutants, which survive until the metamorphosis stage due to maternally supplied transcripts, have small brains and absent imaginal discs (Bandura et al. 2005), the structures that give rise to the legs, wings, antennae, and other external structures—a phenotype reminiscent of MMS despite the vast phylogenetic distance between fly and human. Fourth, we identified numerous components of the prereplication and replication fork complexes that are highly co-expressed with *DONSON* in multiple species spanning from fly to human. Remarkably, our co-expression analysis identified five of the seven known MGS genes (all except *ORC2* and *GMNN*), and *GMNN* (geminin), which was not identified in the co-expression analysis, is a genetic enhancer of the *humpty dumpty* phenotype in fly double mutants (Bandura 2005). Fifth, at the protein level Hd interacts with Asf1, a histone chaperone associated with the MCM2-7 replication helicase.

Mechanistically, it is not unexpected that impaired genome replication due to the reduction of DONSON leads to impaired cellular proliferation and decreased overall growth, especially of organs such as the brain whose development depends on a high number of progenitor cell mitoses. *DONSON* is essential for normal embryonic and fetal development and its complete absence is lethal, so the survivability of MMS is presumably due to the hypomorphic nature of its allele. Between two-thirds and ∼90% of *DONSON* transcripts are not properly spliced in MMS, suggesting that additional MPD cases, particularly MGS-like syndromes, might be caused by other *DONSON* mutations that are less severe.

Indeed, during review of this manuscript, Reynolds et al. (2017) published a series of cases of recessive, hypomorphic mutations in *DONSON* causing primary microcephaly, microcephaly with short stature, and MPD. Most of the phenotypes they report are nonlethal and milder than MMS, implying that the mutations had milder effects on DONSON function than the MMS mutation. However, they also reported one family (P21) from Saudi Arabia that resembles the phenotype of MMS in the First Nations population and which, remarkably, contains the same homozygous mutation, suggesting that a range of hypomorphic *DONSON* mutations cause a wide spectrum of MPD and microcephalic syndromes. Reynolds et al. (2017) further present extensive cell biological evidence for an essential role for DONSON in the replication fork mediating genome replication. Altogether, the unique MMS cases we have studied and the above related syndromes define *DONSON* as a novel human disease gene and will help inform future investigations of its essential function in cellular replication and organismal development.

## Methods

### Human subjects and samples

All human studies were reviewed and approved by the Research Ethics Committee of the University of Saskatchewan and the Committee on Clinical Investigation of Boston Children's Hospital. The study was also supported throughout the project by the Northern Medical Services of the University of Saskatchewan and leaders of the local First Nations community. See Supplemental Methods for further details. All samples in the study are listed in Supplemental Data 1.

### Linkage analysis

Multipoint parametric linkage analysis was performed using Illumina Omni 2.5 SNP array data analyzed with Plink (Purcell et al. 2007) and Merlin (Abecasis et al. 2002) under a recessive model. See Supplemental Methods for full details.

### Genome sequencing

Targeted-capture sequencing was performed using a Roche NimbleGen custom 385K capture array. Paired-end sequencing libraries were generated after capture and sequenced on an Illumina sequencer. Whole-exome sequencing libraries were prepared using the Sure-Select Human All Exon v2 kit (Agilent) and sequenced on an Illumina sequencer. Whole-genome sequencing was performed by Complete Genomics. Targeted-capture and whole-exome data were analyzed using GATK (Van der Auwera et al. 2013). Whole-genome sequencing data were analyzed using Complete Genomics software. Variants were annotated with ANNOVAR (Wang et al. 2010) and filtered if their allele frequency was ≥1% in any of the following public variant databases: ExAC (Lek et al. 2016), 1000 Genomes Project (The 1000 Genomes Project Consortium 2015), NHLBI Exome Sequencing Project (Tennessen et al. 2012), and Complete Genomics 69 control genomes (Drmanac et al. 2010). See Supplemental Methods for further details.

### RNA sequencing

RNA-sequencing libraries were prepared with the Illumina TruSeq Stranded mRNA kit and sequenced on HiSeq 2000 Illumina sequencers. Reads were aligned to the human reference genome (GRCh38/hg38) with HISAT2 (Kim et al. 2015) using standard settings. See Supplemental Methods for full details.

### RT-PCR validation and relative splicing quantification

Residual DNA was eliminated from RNA samples using TURBO DNA-free (Ambion), and cDNA was synthesized using oligo-dT primers. RT-PCR was performed with primers in exon 6 and exon 7, flanking intron 6. The exon 7 primer was labeled with FAM to allow RT-PCR product quantification and calculation of percentage unspliced product on a 3730 DNA Analyzer capillary electrophoresis instrument (Applied Biosystems). See Supplemental Methods for full details.

### In situ hybridization in human and mouse embryos

Human embryo and fetus sections were obtained by the Joint MRC/Wellcome Trust (Grant #099175/Z/12/Z) Human Developmental Biology Resource (www.hdbr.org). In situ hybridization probes were generated by PCR-cloning *DONSON* genomic sequence into plasmids for in vitro transcription with digoxigenin-UTP. Hybridized probes were visualized using anti-digoxigenin alkaline phosphatase-conjugated antibody and NBT/BCIP (Roche). Specificity of the in situ hybridization was confirmed with sense probes. See Supplemental Methods for full method details.

### Knockout mice

Mice heterozygous for a *Donson* knockout allele (*Donson*^tm1a(EUCOMM)Wtsi^) were produced as part of the European Conditional Mouse Mutagenesis Program (EUCOMM) and the International Knockout Mouse Consortium and phenotyped at the Wellcome Trust Sanger Institute. See Supplemental Methods for further details.

### siRNA knockdown and qPCR

cDNA of HeLa cells transfected with *DONSON* or *GFP* siRNA was assayed using a qPCR panel of 91 cell cycle regulation genes (Roche) on a LightCycler 480 instrument (Roche). cDNA of MMS patient samples and controls was assayed using 18S rRNA and *DONSON* TaqMan qPCR assays (Thermo Fisher). See Supplemental Methods for full method details.

## Data access

DNA and RNA sequencing data from this study (all samples except HI-1400 and HI-2185) have been submitted to the NCBI Database of Genotypes and Phenotypes (dbGaP; https://www.ncbi.nlm.nih.gov/gap) under accession number phs000492.v2.p1. Data for Autism Genetic Resource Exchange (AGRE) cell lines (HI-1400 and HI-2185) have been submitted to the AGRE data repository (https://research.agre.org). Sanger sequencing from this study has been submitted to the NCBI Trace Archive (https://trace.ncbi.nlm.nih.gov/Traces/trace.cgi) under TI numbers 2344113440–2344113468.

## Supplementary Material

Supplemental Material
