# Supplementary material for: Integrated genome and transcriptome sequencing identifies a noncoding mutation in the genome replication factor DONSON as the cause of microcephaly-micromelia syndrome
Source: Genome Res. 2017 Aug;27(8):1323–35. doi: 10.1101/gr.219899.116 (PMC5538549; doi:10.1101/gr.219899.116)
Supplement: Supplemental Material [file supp_27_8_1323__index.html]

Integrated genome and transcriptome sequencing identifies a noncoding mutation in the genome replication factor DONSON as the cause of microcephaly-micromelia syndrome — Supplemental Material 

# Integrated genome and transcriptome sequencing identifies a noncoding mutation in the genome replication factor *DONSON* as the cause of microcephaly-micromelia syndrome

## Supplemental Material

undefined

- Supplemental\_Materials.pdf
- Supplemental\_Data\_1.xlsx
- Supplemental\_Data\_2.xlsx
