# Supplementary material for: Integrated genome and transcriptome sequencing identifies a noncoding mutation in the genome replication factor DONSON as the cause of microcephaly-micromelia syndrome
Source: Genome Res. 2017 Aug;27(8):1323–35. doi: 10.1101/gr.219899.116 (PMC5538549; doi:10.1101/gr.219899.116)
Supplement: Supplemental Material [file supp_gr.219899.116_Supplemental_Materials.pdf]

Supplemental Materials for:

Integrated genome and transcriptome sequencing identifies a non-coding mutation in the genome replication factor *DONSON* as the cause of microcephaly-micromelia syndrome

Gilad D. Evrony¶, Dwight R. Cordero¶, Jun Shen¶, Jennifer N. Partlow, Timothy W. Yu, Rachel E. Rodin, R. Sean Hill, Michael E. Coulter, Anh-Thu N. Lam, Divya Jayaraman, Dianne Gerrelli, Diana G. Diaz, Chloe Santos, Victoria Morrison, Antonella Galli, Ulrich Tschulena, Stefan Wiemann, M. Jocelyne Martel, Betty Spooner, Steven C. Ryu, Princess C. Elhosary, Jillian M. Richardson, Danielle Tierney, Christopher A. Robinson, Rajni Chibbar, Dana Diudea, Rebecca Folkerth, Sheldon Wiebe, A. James Barkovich, Ganeshwaran H. Mochida, James Irvine, Edmond G. Lemire, Patricia Blakley, Christopher A. Walsh*

¶ These authors contributed equally to this work.
* Corresponding author: christopher.walsh@childrens.harvard.edu

Table of Contents

Supplemental Figure 1……………………………………………………………… 2

Supplemental Figure 2……………………………………………………………… 4

Supplemental Figure 3……………………………………………………………… 6

Supplemental Figure 4……………………………………………………………… 8

Supplemental Figure 5……………………………………………………………… 10

Supplemental Data Legends………………………………………………………… 12

Supplemental Methods……………………………………………………………… 13

Supplemental References…………………………………………………………… 18

Supplemental Fig. 1

A

Individual	Sequencing method	Full ROH 0x	Full ROH ≥ 1x	Full ROH ≥ 10x	Full ROH ≥ 20x	Full ROH ≥ 30x	ROH coding exons 0x	ROH coding exons ≥ 1x	ROH coding exons ≥ 10x	ROH coding exons ≥ 20x	ROH coding exons ≥ 30x
**412**	**Whole-exome seq**	N/A	N/A	N/A	N/A	N/A	1.4%	98.6%	97.5%	96.3%	95.4%
**13001**	**Whole-exome seq**	N/A	N/A	N/A	N/A	N/A	0.9%	99.1%	96.5%	95.0%	91.0%
**Whole-exome seq average**	**Whole-exome seq**	N/A	N/A	N/A	N/A	N/A	1.1%	98.9%	97.0%	95.7%	93.2%
**13001**	**Targeted-capture seq**	7.2%	92.8%	82.7%	80.5%	79.2%	0.0%	100.0%	99.8%	99.6%	99.3%
**12601**	**Whole-genome seq**	0.4%	99.6%	98.3%	95.2%	86.6%	0.9%	99.1%	97.9%	91.6%	78.9%

B

		Coding exon frameshift		Coding exon nonsense		Coding exon missense		Coding exon synonymous		Splice site		Total coding or splice site		Structural variants	
	**Public database filtering:**	-	+	-	+	-	+	-	+	-	+	-	+	-	+
**Individual**	**Sequencing method**														
**412**	**Whole-exome seq**	2	0	0	0	3	0	2	0	0	0	**7**	**0**	N/A	N/A
**13001**	**Whole-exome seq**	2	0	0	0	3	0	2	0	0	0	**7**	**0**	N/A	N/A
**13001**	**Targeted-capture seq**	2	0	0	0	3	0	2	0	0	0	**7**	**0**	0	0
**12601**	**Whole-genome seq**	2	0	0	0	3	0	2	0	0	0	**7**	**0**	0	0

		Non-coding RNA exon		UTR		Intron		± 1 kb of transcript start/stop		Other intergenic		Total non-coding	
	**Public database filtering:**	-	+	-	+	-	+	-	+	-	+	-	+
**Individual**	**Sequencing method**												
**412**	**Whole-exome seq**	0	0	0	0	22	3	2	0	0	0	**24**	**3**
**13001**	**Whole-exome seq**	1	0	0	0	32	2	2	0	15	1	**50**	**3**
**13001**	**Targeted-capture seq**	4	0	11	0	263	14	15	0	164	18	**457**	**32**
**12601**	**Whole-genome seq**	4	0	14	0	305	12	17	0	176	6	**516**	**18**

C

Number of variants (all types) in ROH

Pre-filtering

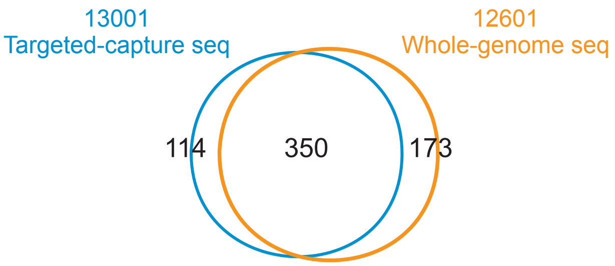

Post-filtering

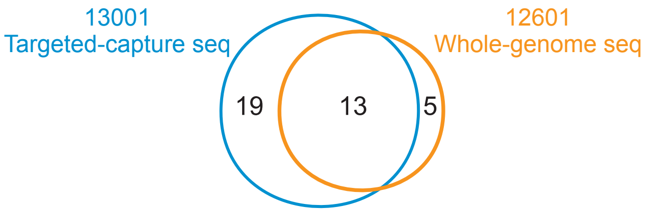

D

Coding or splice site variants in ROH

Pre-filtering

Chr	Position	Ref allele	Variant allele	Gene	Effect	Detected? 412 WES	13001 WES	13001 targeted-capture	12601 WGS
21	33524806	C	T	GART	Missense	+	+	+	+
21	33552836	C	T	SON	Missense	+	+	+	+
21	33553954	C	T	SON	Missense	+	+	+	+
21	33576378	-	A	SON	Frameshift	+	+	+	
21	33576379	-	A	SON	Frameshift				+
21	33576391	A	-	SON	Frameshift	+	+	+	+
21	33829643	C	T	ITSN1	Synonymous	+	+	+	+
21	33912379	A	G	ATP5O	Synonymous	+	+	+	+

Supplemental Figure 1. Genome coverage and concordance of variant detection among different DNA sequencing methods.
(A) Fraction of the minimal region of homozygosity (ROH) interval linked to MMS sequenced at different read depths (0x, ≥ 1x, ≥ 10x, ≥ 20x, ≥ 30x), for each DNA sequencing method. Coverage statistics are shown for both the full ROH (including introns and intergenic regions) and for only coding exons in the ROH (RefGene coding exons plus their 2 bp intronic flanks that would be candidate splice variants).
(B) Number of homozygous variants identified in the MMS minimal region of homozygosity (ROH), categorized by variant type, both before (-) and after (+) filtering out variants found at ≥ 1% allele frequency in public variant databases (see Methods). Splice site variants are intronic variants within 2 bp of the intron-exon junction. UTR: untranslated regions; ± 1 kb of transcript: variants within 1 kb of transcript start or stop sites. See **Supplemental Data 2** for a full listing of the variants identified after filtering.
(C) Venn diagrams showing concordance of detection of the variants of all types in the MMS ROH with targeted and whole-genome sequencing, both before (-) and after (+) filtering out variants found at ≥ 1% allele frequency in public variant databases (see Methods).
(D) Table showing concordance of detection of coding region or splice site variants (i.e. variants in RefGene coding exons or their 2 bp intronic flanks) in the MMS ROH by whole-exome sequencing (WES), targeted-capture sequencing, and whole-genome sequencing (WGS), before filtering out variants found at ≥ 1% allele frequency in public variant databases. After filtering, there were no coding region or splice site variants detected by any of the sequencing methods.

Supplemental Fig. 2

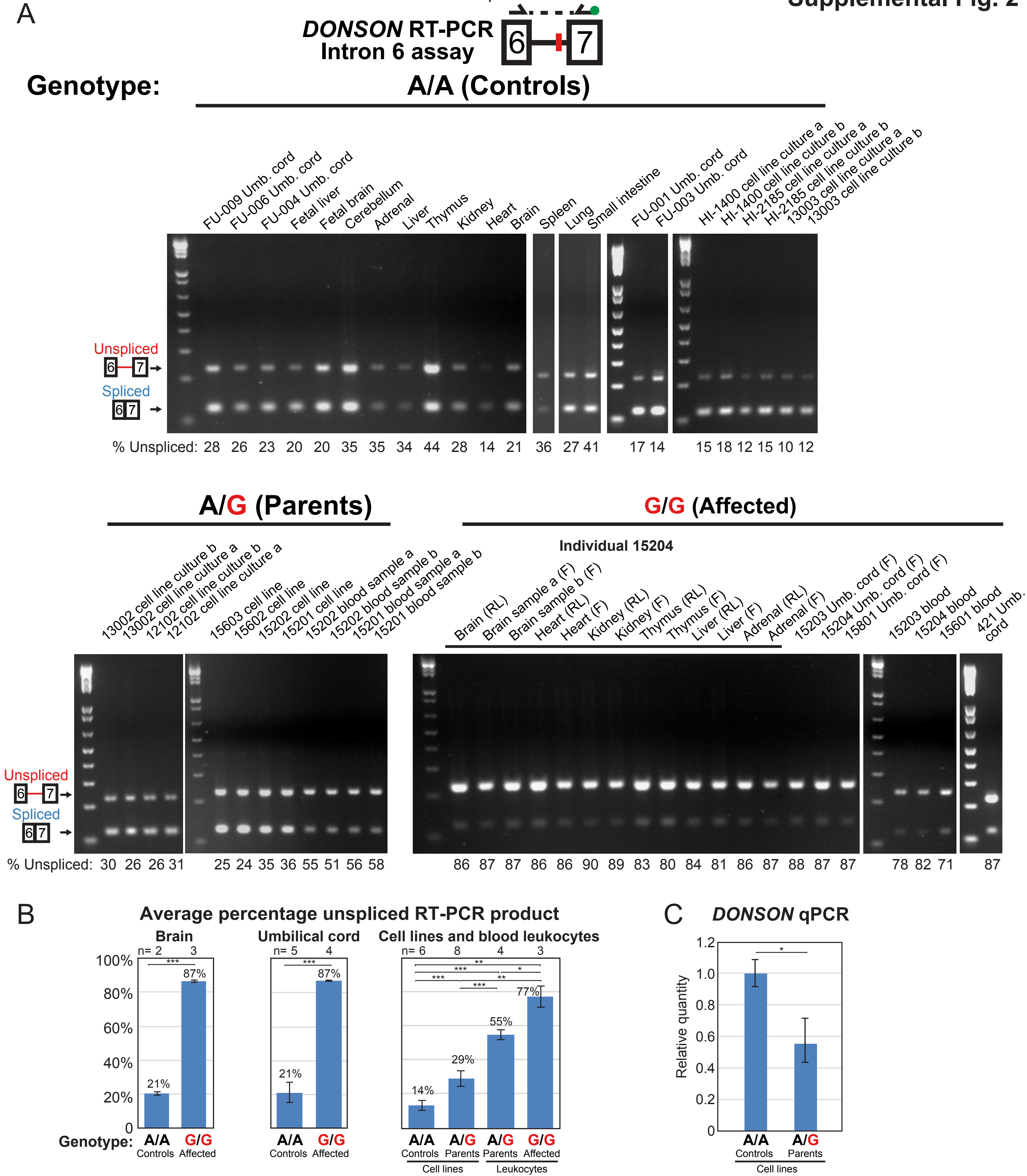

Supplemental Figure 2. RT-PCR assay of the *DONSON* intron retention splice defect in MMS.

(A) RT-PCR spanning from exon 6 to exon 7 of *DONSON* in various tissues shows increased retention of intron 6 in MMS samples, which are homozygous for the c.1047-9A>G (Chr21:33582064 T>C) mutation, compared to heterozygous parents and healthy wild-type controls (unspliced transcript with intron 6: 230 bp; spliced transcript: 121 bp). A subset of this data is shown in **Fig. 3E**. Umbilical cord is abbreviated Umb. cord. RT-PCR was performed with the PCR primer for exon 7 containing a FAM-fluorescent label allowing quantification of the RT-PCR products with a capillary electrophoresis DNA analyzer (see Methods). Percentage unspliced RT-PCR product was calculated as [Area of unspliced band] / [Area of unspliced band + Area of spliced band]. See **Fig. 3F** for measurements averaged across all samples of each genotype. Note that the quantification performed by the DNA analyzer of the fluorescent primer does not correspond to the intensity seen on the electrophoresis gel. The former is a more accurate measure of the amounts of each PCR product since it quantifies each individual DNA molecule equally regardless of its size, since each contains a single FAM-labeled primer, whereas the ethidium bromide-stained gel gives a larger signal for a large DNA molecule (i.e. unspliced product) than a small DNA molecule (i.e. spliced product). Therefore, on the gel the relative intensity of unspliced versus spliced product is greater than the relative molar amounts of unspliced versus spliced products that are quantified by the DNA analyzer. Note also that the RT-PCR assay can evaluate relative splicing differences between samples but it is not an absolute measure of splicing, since the PCR amplification efficiencies of the unspliced and spliced products differ.

(B) Percentage unspliced RT-PCR product by genotype, calculated as in **Fig. 3F** and **Supplemental Fig. 2A** for three representative tissue types: whole brain (fetal and adult) [left], umbilical cord [middle], and cell lines and blood leukocytes [right]. All group comparisons were statistically significant (*: $p < 0.05$; **: $p < 0.005$; ***: $p < 0.0005$; two-tailed unpaired *t*-tests with Holm multiple comparisons adjustment). See **Fig. 3F** for percentage unspliced RT-PCR product averaged across all samples of each genotype (i.e. including other tissue types such as heart, kidney, liver, etc.).

(C) qPCR shows decreased *DONSON* transcript levels in heterozygous MMS cell lines (derived from parents of affected patients; n=6 cell lines) versus control cell lines (n=3), suggesting nonsense-mediated decay. Error bars are 95% confidence intervals. Note that cell lines from affected newborns could not be derived, since they fail to proliferate. Each qPCR reaction was performed in triplicate (technical replicates) with 18S rRNA as an endogenous control.

Supplemental Fig. 3

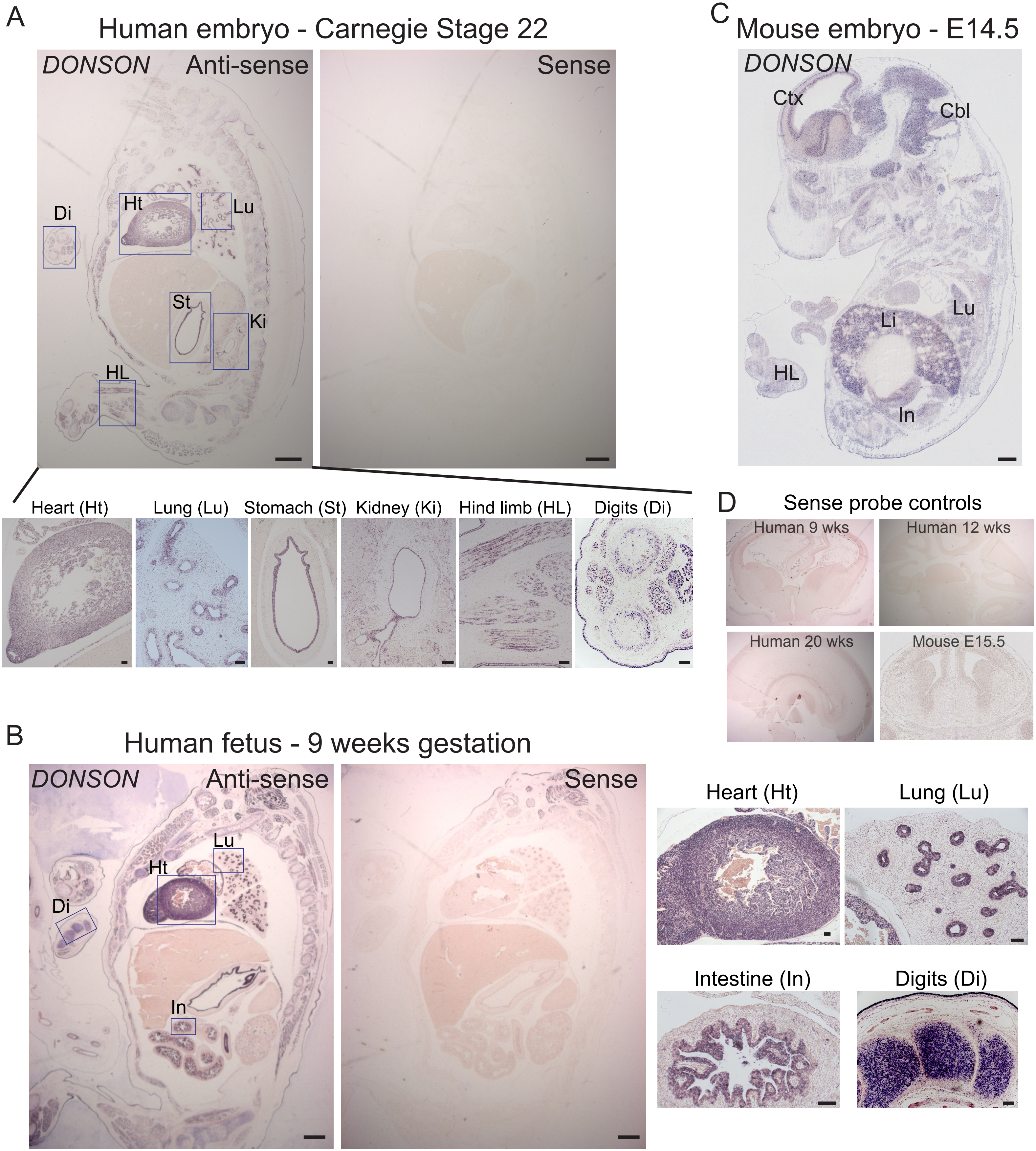

Supplemental Figure 3. *DONSON* expression in human and mouse prenatal development.
(A) *DONSON* expression by in situ hybridization in a sagittal section of a Carnegie stage 22 (about 7.5 weeks gestation) human embryo (head removed). Expression is evident in numerous locations, including heart (Ht), lung (Lu), stomach (St), kidney (Ki), hind limb (HL), and forelimb digits (Di). Hybridization was performed with antisense probe 1 (left) (see Supplemental Methods); low signal with sense probe 1 (right) confirms specificity of the staining. Scale bars for main images (top) are 1000 μm; scale bars for smaller zoomed images (bottom) are 100 μm.
(B) *DONSON* expression by in situ hybridization in a sagittal section of a 9-week gestation human fetus (head removed). Expression is evident in numerous locations, including heart (Ht), lung (Lu), intestine (In), and forelimb digits (Di). Hybridization was performed with antisense probe 2 (left) and sense probe 2 (right). Scale bars for main images (left) are 1000 μm; scale bars for smaller zoomed images (right) are 100 μm.
(C) *DONSON* expression by in situ hybridization in a sagittal section of an E14.5 mouse embryo, obtained from GenePaint (www.genepaint.org; accession AF193608) (Visel et al. 2004). Expression is evident in the neocortex (Ctx), cerebellum (Cbl), lung (Lu), liver (Li), hindlimb (HL), and intestine (In). Other sections (not shown) show expression in forelimb, kidney, spinal cord vertebrae, and other regions. Scale bar 500 μm.
(D) Control *DONSON* sense sequence probe in situ hybridizations for human 9, 12, and 20 weeks-gestation (wks) fetal brain sections, and mouse E15.5 brain sections, corresponding to antisense probe hybridizations in **Figures 4A-D** (adjacent sections). Sense probe hybridizations show negligible signal, confirming specificity of the antisense probe staining.

Supplemental Fig. 4

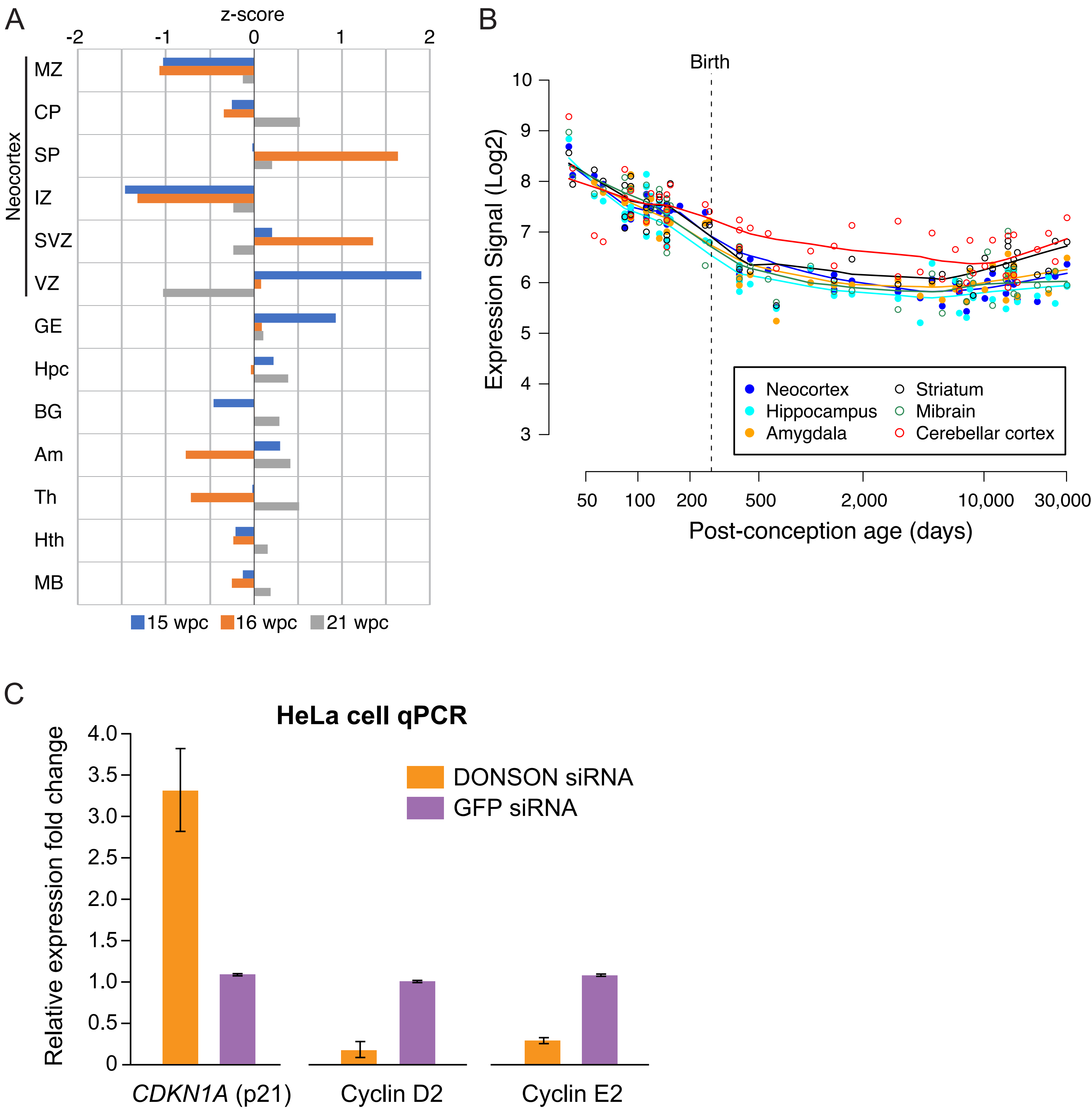

Supplemental Figure 4. *DONSON* expression in human brain development and *DONSON* knockdown effect on cell cycle regulators.
(A) Normalized *DONSON* expression levels (z-scores) in different laser-microdissected regions of 15-, 16-, and 21-weeks post-conception (wpc) human fetal brains, from microarray data of the BrainSpan Atlas of the Developing Human Brain (Miller et al. 2014). In the neocortex, the peaks of *DONSON* expression are in the ventricular zone at 15 wpc, the subventricular zone and subplate at 16 wpc, and the cortical plate at 21 wpc. MZ, marginal zone; CP, cortical plate; SP, subplate; IZ, intermediate zone; SVZ, subventricular zone; VZ, ventricular zone; GE, ganglionic eminence; Hpc, hippocampus; BG, basal ganglia; Am, amygdala; Th, thalamus; Hth, hypothalamus; MB, midbrain.
(B) Graph of *DONSON* expression levels across brain regions in prenatal through adult time points obtained from the Human Brain Transcriptome project (http://www.hbatlas.org) (Kang et al. 2011). Solid lines trace the estimated trajectories across time points. *DONSON* expression is highest in the prenatal period and then plateaus to a lower level. Expression levels were determined in this project using Affymetrix GeneChip microarray.
(C) *DONSON* siRNA knockdown in HeLa cells leads to increased *CDKN1A* (p21) and decreased cyclin D2 and cyclin E2 transcript levels as measured by qPCR, relative to control green fluorescent protein (GFP) siRNA, consistent with impaired progression through the G1/S transition of the cell cycle. Error bars are standard deviation of three technical replicates each of two siRNA knockdown biological replicates.

Supplemental Fig. 5

A

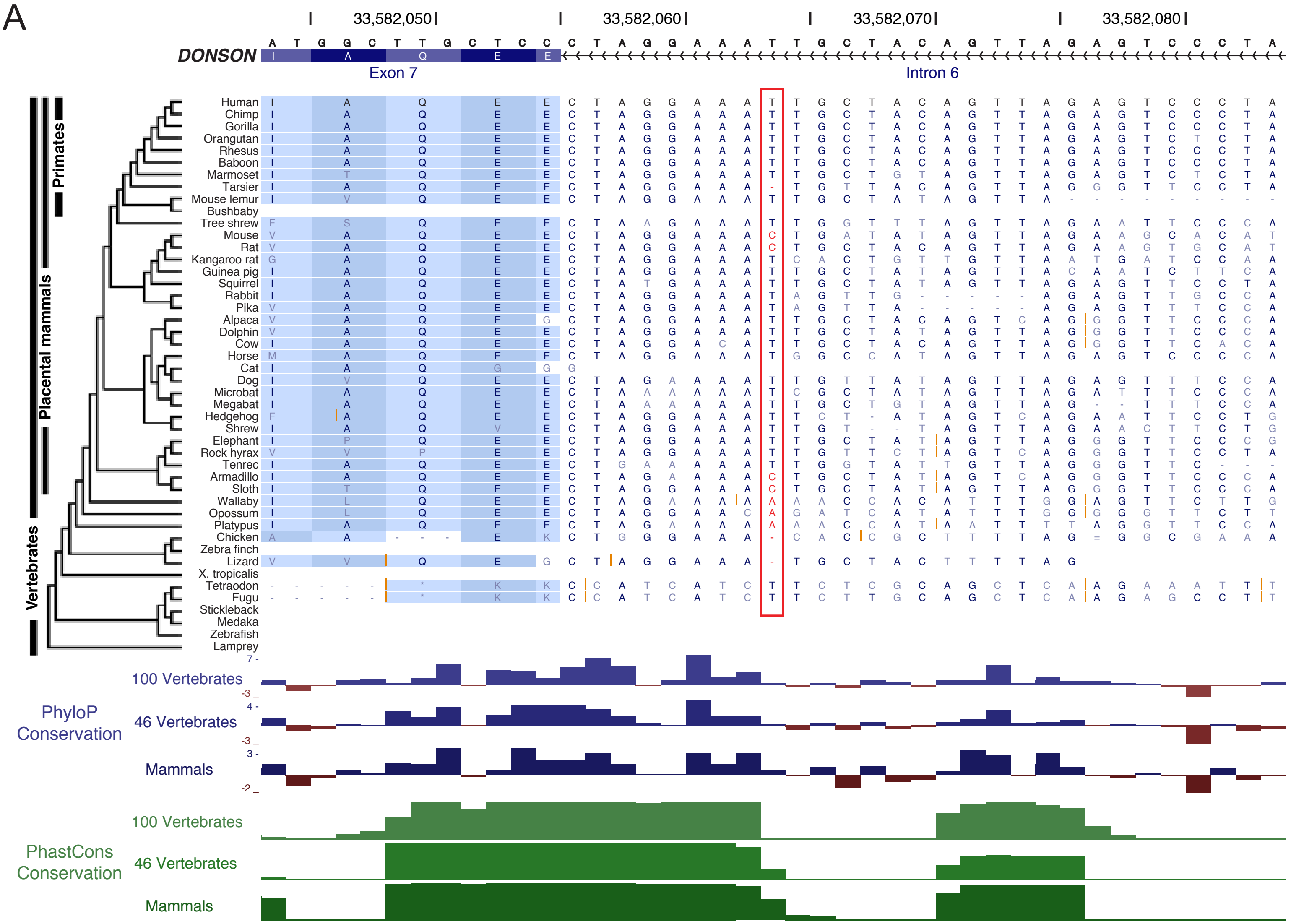

B

Species	Sequence accession	Length (AA)	% Identity vs. H. Sapiens	Blast E-value vs. H. Sapiens
H.sapiens (human)	NP_060083.1	566		
P.troglodytes (chimpanzee)	NP_001233483.1	566	99	0
M.mulatta (macaque)	XP_001091031.2	564	95	0
C.lupus (dog)	XP_005638908.1	569	85	0
B.taurus (cow)	NP_001076914.1	558	82	0
M.musculus (mouse)	NP_068366.1	560	76	0
R.norvegicus (rat)	XP_006248109.1	559	76	0
G.gallus (chicken)	XP_416713.4	559	67	0
X.tropicalis (frog)	NP_989384.1	577	65	0
D.melanogaster (fly)	NP_649531.1	568	34	6 E-53
A.gambiae (mosquito)	XP_315901.4	609	33	1 E-50
C.elegans (roundworm)	NP_001254011.1	594	20	8 E-6
A.thaliana (plant)	ANM65041.1	590	26	2 E-8
M.elongata (fungus)	OAQ33768.1	583	24	2 E-14

Supplemental Figure 5. Evolutionary conservation of *DONSON* and the *DONSON* MMS variant.

(A) Multi-species alignment of the *DONSON* intron 6-exon 7 junction. The sequence and coordinates above the transcript ideogram are from the human reference genome (GRCh38/hg38). The red rectangle highlights the site of the pathogenic mutation in MMS (Chr21:33,582,064 T>C; c.1047-9A>G), and red bases at are nucleotides that differ from the human sequence. Note that mouse, rat, armadillo, sloth, and other species not shown here such as golden hamster, prairie vole, and cape elephant shrew, have a “C” at the corresponding position of the MMS mutation, the same base as the MMS disease-causing variant, and several other species have a “G” or “A”, highlighting that conservation analyses alone may sometimes not be sufficient in predicting non-coding mutation pathogenicity. Orange lines in alignments indicate gaps in the human sequence relative to the species sequence, and dashes indicate gaps in the species sequence relative to the human sequence. PhyloP (Pollard et al. 2010) and PhastCons (Siepel et al. 2005) phylogenetic conservation scores at the bottom show a peak of conservation extending into intron 6, until 1 base prior to the pathogenic *DONSON* mutation site. PhyloP and PhastCons conservation calculated across a 100-vertebrate species alignment (including 54 additional species not shown here) are also displayed. The figure was generated with the UCSC genome browser (Kent et al. 2002) using the hg19 Multiz 46-vertebrate and Multiz 100-vertebrate alignment and conservation tracks. The phylogenetic tree at the left is from the UCSC genome browser hg19 46-vertebrate alignment track. Zebra finch, stickleback, medaka, zebrafish, and lamprey do not have alignable sequence at the displayed locus. However, all 100 vertebrate species from the Multiz 100-vertebrate track have at least some part of *DONSON* in their genomes, except for white-throated sparrow and zebrafish that do not due to low-quality incomplete genome assemblies rather than absence of *DONSON* from their genomes (data not shown; see UCSC genome browser for full list of the 100 vertebrate species).

(B) *DONSON* is conserved across multi-cellular eukaryotes. Representative *DONSON* orthologues found in a search with the human *DONSON* protein sequence using BLASTP (Altschul et al. 1990) with E-value < 10^{-5} (expected number of chance matches in searching the database; a lower E-value indicates higher statistical significance of the match). Percent protein sequence identity of orthologous proteins versus human *DONSON* are shown. No matches to eubacteria, archaea, or unicellular eukaryotes were found at the given E-value threshold.

Supplemental Data Legends

Supplemental Data 1. Individuals and samples profiled in this study.
List of all the samples in this study and the experiments performed on each sample. Samples are grouped by nuclear family (**Fig. 1E**). Affected individuals are highlighted in bold text and gray shading. Control samples are listed at the bottom. Birth weight, head circumference, and length at birth with z-score (number of standard deviations from the mean for the gestational age) are shown when available for affected individuals. Note that the summary statistics described in the main text for these measurements included additional individuals that were not genetically profiled in this study. The number of sequencing reads in millions (M) or total sequencing coverage in gigabases (Gb) are shown for sequencing experiments.

Supplemental Data 2. Variants detected by each DNA sequencing method.
List of all homozygous variants found by whole-exome, targeted-capture, or whole-genome sequencing in the minimal region of homozygosity (ROH) linked to MMS (Chr21:33,364,965-34,029,433; GRCh38/hg38). Variants with allele frequency ≥ 1% in one of the public variant databases (see Methods) were filtered and are not shown. Allele depth and genotype quality columns are blank if the variant was not detected in the sample. The pathogenic mutation causing MMS is highlighted in gray. Start and end positions are the first and last bases of the variant (GRCh38/hg38; 1-base closed numbering). Insertions and deletions are annotated with '-' in the reference sequence and variant sequence columns, respectively. Read depths are the number of reads at the position, excluding reads that were considered uninformative by the GATK pipeline (uninformative reads are those that do not provide enough statistical evidence to support one allele over another; see GATK documentation for details). Note that for one variant (21:33680692-33680697, 13001 Targeted-capture), read depths are 0 because the genotype was called by GATK using only non-informative reads. Allele frequencies in public variant databases are shown: 1) ExAC, Exome Aggregation Consortium; 2) 1,000 Genomes Project; 3) ESP, NHLBI Exome Sequencing Project; 4) Complete Genomics control genomes. dbSNP identifiers are shown for dbSNP build 142.

Supplemental Methods

Human subjects and samples

All human studies were reviewed and approved by the Research Ethics Committee of the University of Saskatchewan and the Committee on Clinical Investigation of Boston Children's Hospital. Biological samples and medical information were collected from the affected individuals and their family members after obtaining written informed consent. The study was also supported throughout the project by the Northern Medical Services of the University of Saskatchewan and leaders of the local First Nations community.

Z-scores of weight, head circumference, and length for each gestational age were calculated using the PediTools Growth Calculator (http://peditools.org/fenton2013).

DNA was extracted from blood samples and cell lines using the DNeasy and MagAttract DNA Blood kits (Qiagen). Lymphoblastoid cell lines of individuals from the studied families were created from blood samples by Epstein-Barr virus transformation (Partners Healthcare BioBank). Additional control cell lines of neurologically normal individuals (HI-1400 and HI-2185) were obtained from the Autism Genetic Research Exchange (AGRE). Umbilical cords from normal newborns were obtained by Dr. Jonathan Hecht (Department of Pathology, Beth Israel Deaconness Medical Center). DNA was obtained and extracted from cheek swab samples using the Oragene kit (OraSure Technologies). Peripheral blood RNA was collected and extracted using the PAXgene Blood RNA kit (Qiagen). Tissue samples from affected newborns were preserved after autopsy using either RNAlater (Ambion) or by rapid freezing to -80° C. A panel of DNA from neurologically normal Caucasian individuals was obtained from Coriell: plates NDPT020 and NDPT021 (184 individuals) were profiled by Sanger sequencing and plates NDPT019, NDPT022, NDPT023, NDPT024, NDPT079, and NDPT084 (552 individuals) were profiled by Sequenom genotyping for the presence or absence of the MMS *DONSON* variant.

Tissues were homogenized using either a mortar and pestle on dry ice or with the TissueLyser II instrument (Qiagen). RNA was extracted from tissue homogenates with the mirVana kit (Ambion); cell line samples were lysed directly using lysis buffer from the mirVana kit. Control human tissue RNA was obtained from Clontech. RNA quality was assessed with the Agilent 2100 Bioanalyzer. All samples in the study are listed in **Supplemental Data 1**.

Linkage analysis

Multipoint parametric linkage analysis was performed using Illumina Omni 2.5 v1.1 SNP array data from 7 affected (402, 421, 12101, 12601, 13001, 15203, and 15601) and 15 unaffected (401, 416, 417, 422, 9601, 9602, 12102, 12103, 12602, 12603, 13002, 15201, 15202, 15602, and 15603) individuals (**Supplemental Data 1**). SNP arrays were also run on affected individuals 419 and his mother 418, but both were excluded from the linkage analysis because the SNP call rate for 419 was below the 99% quality control threshold. As it was known that the individuals in the study were related, but the exact relationships were not known, each nuclear family was analyzed independently, and the parents were assumed to be first cousins in order to generate a conservative LOD score. Plink version 1.07 (Purcell et al. 2007) was used to clean the data by removing from analysis poor performing SNPs with >5% NoCall or a minor allele frequency < 10% in a set of 343 individuals who were genotyped on the same array under similar conditions.

SNPs were also removed by Plink from the analysis if, among the 22 individuals genotyped for this study, they had a NoCall in any individual, were homozygous among all individuals, or contained a Mendelian error. Finally, a set of unaffected and unrelated individuals genotyped on the same array under similar conditions were used to remove SNPs not in Hardy-Weinberg equilibrium ($p < 1x10^{-8}$) and to prune the remaining SNPs to a set that were approximately in linkage equilibrium (using the Plink indep command with a window size of 50, step of 5, and variance inflation factor of 1.1). Merlin version 1.1.2 (Abecasis et al. 2002) was also used to remove SNPs with a non-Mendelian error. The final data set consisted of 51,125 SNPs. Merlin was then used to calculate LOD scores assuming a disease allele frequency of 1/10,000, 100% disease penetrance under a recessive model, and equal allele frequency among all SNPs. The LOD interval associated with the disease was calculated as the interval with LOD > Z_{max}-3, where Z_{max} is the maximum LOD score in the interval. Older samples (412, 414, 420) were run previously on Affymetrix 5.0 SNP arrays and were also homozygous in the interval (data not shown), but this data was not used for LOD score calculation that analyzed only samples profiled by Illumina Omni SNP arrays.

Genome sequencing

Targeted-capture sequencing was performed using a Roche NimbleGen custom 385K capture array that densely tiled capture probes across the region Chr21:33,165,611-34,718,221 (GRCh38) encompassing the entire ROH (coding and non-coding regions). Paired-end sequencing libraries were generated after capture and sequenced on an Illumina sequencer. Whole-exome sequencing libraries were prepared using the Sure-Select Human All Exon v2.0 kit (Agilent) and sequenced on an Illumina sequencer at the Broad Institute (Cambridge, MA). Whole-genome sequencing was performed by Complete Genomics. Targeted-capture and whole-exome data were analyzed using the GATK Best Practices pipeline (Van der Auwera et al. 2013) aligned to the GRCh37 human reference genome, and variant calls were annotated with ANNOVAR (Wang et al. 2010). Whole-genome sequencing data was analyzed using Complete Genomics software version 1.11 with the GRCh37 human reference genome, and variant calls were converted to VCF files (using the Complete Genomics masterVar2VCF script) and annotated with ANNOVAR. The location of variants relative to genomic elements (**Table 1**) (e.g. exonic, intronic, UTR, etc.) was determined by ANNOVAR using the RefGene database (NCBI reference genes). Variants were filtered for those called as homozygous by the analysis pipeline, and whole-exome and targeted-capture variants (analyzed by the GATK pipeline) were filtered for genotype quality > 10.

Variants were annotated with ANNOVAR for allele frequencies in control populations using the following databases: 1) Exome Aggregation Consortium (ExAC, 60,706 individuals; http://exac.broadinstitute.org)(Lek et al. 2016); 2) 1,000 Genomes Project (http://www.1000genomes.org) (The 1000 Genomes Project Consortium 2015); 3) NHLBI Exome Sequencing Project (6,503 individuals; http://evs.gs.washington.edu/EVS)(Tennessen et al. 2012); 4) Complete Genomics control genomes (69 individuals; http://www.completegenomics.com/public-data/69-genomes)(Drmanac et al. 2010). Variants were filtered if their allele frequency was ≥ 1% in any one of these public variant databases, since the pathogenic mutation of MMS would be expected to be significantly more rare in the population. Finally, variant coordinates were converted from GRCh37 to GRCh38 with the UCSC Genome Browser LiftOver tool.

Array comparative genomic hybridization and structural variant analysis

Array comparative genomic hybridization analysis of copy number variation was performed with DNA samples from two affected individuals (12101 and 12601) and one unaffected parent (12103) using custom Roche NimbleGen arrays. The array contained probes tiled at low density across all of Chromosome 21 and at high density every 10 bp and every 50 bp across the region of interest including the entire ROH. Copy number variation analysis of the array data was performed by searching for contiguous probes with increased or decreased signal after normalizing to control and parent signals.

Structural variant analysis of the targeted-capture sequencing data was performed by searching for clusters of paired-end reads that either: 1) align in the wrong orientation; i.e. the reads of a pair align in the same direction (implying an inversion) or point away from each other (implying a duplication), rather than the expected orientation pointing towards each other; 2) each read of a pair aligns to a different chromosome (implying a translocation); 3) reads of a pair align in the correct orientation but with an insert size greater than 3 standard deviations from the average insert size (implying a deletion); or 4) only one of the reads of a pair aligned to the genome (implying an insertion). Structural variant analysis of the whole-genome sequencing data was obtained from the output of the Complete Genomics software version 1.11 analysis of the data.

RNA sequencing

RNA-sequencing libraries were prepared with the Illumina TruSeq Stranded mRNA kit that preserves RNA strand information. Paired-end reads (50 or 51 bp x 2) were sequenced on HiSeq 2000 Illumina sequencers (Harvard Biopolymers Facility and Expression Analysis). Reads were aligned to the human reference genome (GRCh38/hg38) with HISAT2 v.2.0.4 (Kim et al. 2015) using standard settings.

Intron retention of each intron of *DONSON* (**Figure 3B**) was estimated by pooling all reads from all the samples of a given genotype (i.e. wild-type controls, heterozygous parents, and homozygous individuals) that were part of a concordantly and uniquely aligned read pair (excluding PCR duplicates), and then calculating for each intron the number of reads reflecting intron retention (e.g. for intron 6, exon 6–intron 6 junction-spanning reads plus intron 6–exon 7 junction-spanning reads) divided by the number of intron retention reads plus two times the number of reads reflecting intron splicing (e.g. for intron 6, exon 6–exon 7 splice reads that skip intron 6). The latter term is multiplied by two since each transcript with intron retention would produce on average twice as many intron-exon junction reads (one for each intron-exon junction) as a transcript that spliced out the intron, which would produce one exon-exon junction splice read. The 95% confidence intervals in **Figure 3C** were calculated with the *prop.test* function in R using the read numbers used to estimate intron retention as described above for **Figure 3B**. Gene expression was analyzed with StringTie v.1.3.3 and Ballgown v.2.6.0 with standard settings after removing PCR duplicates (Pertea et al. 2016). Genome-wide differential gene expression analyses were performed for umbilical cord samples (wild-type vs. homozygous MMS mutation) and peripheral blood samples (heterozygous vs. homozygous MMS mutation), but did not yield differentially expressed genes at genome-wide statistical significance due to the small number of available homozygous samples.

RT-PCR validation and relative splicing quantification

Residual DNA was eliminated from RNA samples using TURBO DNA-free (Ambion). cDNA was synthesized from RNA using the SuperScript III First-Strand Synthesis Kit with oligo-dT primers (Invitrogen). Each sample was run in two reactions, one with and one without reverse transcriptase. Downstream PCR experiments with the no-reverse transcriptase controls did not produce any product for any sample, confirming the absence of contaminating DNA. RT-PCR was performed with: 4ul GoTaq Hot Start 5x buffer (Promega), 1.2ul of 25mM $MgCl_2$, 0.4ul dNTP (10mM each, New England BioLabs), 1.4ul of 10uM exon 6 primer (GAAAGTGGCCATAAGAAGGAGA), 1.4ul of 10uM FAM-labeled exon 7 primer (CTTGCACACCCATCTCTTCC), 10.4ul water, 0.2ul GoTaq Hot Start Polymerase (Promega), and 1ul cDNA. Reactions were cycled at: 95°C 5 mins; (95°C 30 sec, 60°C 30 sec, 72°C 30 sec) x 35 cycles; 72°C 8 mins; hold at 10°C. cDNA for each sample was used at a dilution optimized based on the observed intensity of RT-PCR products on gel electrophoresis; too little input cDNA leads to low product yield, and too much input cDNA leads to a hybrid spliced/unspliced product migrating at larger sizes. RT-PCR products were quantified using a 3730 DNA Analyzer capillary electrophoresis instrument (Applied Biosystems). Percentage unspliced RT-PCR product was calculated as [Area of unspliced band] / [Area of unspliced band + Area of spliced band]. Note that due to differing PCR amplification efficiencies of the spliced and unspliced products, this measurement can only be used to evaluate relative splicing differences between samples or sample groups and not as an absolute measure of splicing.

In situ hybridization in human and mouse embryos

Probes were generated by PCR using three sets of primers for the human *DONSON* mRNA transcript. The first PCR primer set amplifies a 219 bp sequence (probe 1) from exon 9 to exon 10 of *DONSON*, using the forward primer CCATGCTGCTCAAATCTTCA and reverse primer TCAGCACCACATTCCGTAAA. The second PCR primer set amplifies a 225 bp sequence (probe 2) from exon 4 to exon 5 of *DONSON*, using the forward primer ACCCTGCTTTGTCTTGGCTA and reverse primer CTAATCCTGCTGCTCGGAAC. The third PCR primer set amplifies a 668 bp sequence (probe 3) from exon 2 to exon 7 of *DONSON*, using the forward primer ACTGAATTACCTCAGACTTCACA and reverse primer GCTTGCTCCTCCCCATATCC. Probe 3 was used for in situ hybridization of embryonic mouse brain (probe sequence identity to mouse transcript is 83%) (**Fig. 4D**). PCR products were purified by gel extraction and cloned into the pGEM®-T Easy vector (Promega). Selected colonies were grown overnight in 200ml LB-Broth (Invitrogen). DNA plasmids were purified with the HiSpeed plasmid Maxiprep kit (Qiagen) Plasmid inserts were sequenced to determine the orientation of the insert. Plasmids were then linearized with SacI for T7 transcription and NcoI for SP6 transcription (Promega). Digestion products were purified and in vitro transcription was performed with incorporation of digoxigenin-UTP using the DIG RNA Labelling Kit (SP6/T7) (Roche) with either the SP6 or T7 polymerase depending on the insert direction. DNAseI (Roche) was used to eliminate the DNA templates. The probes were then cleaned using CHROMA SPIN-100+DEPC-H2O Columns (Takara/Clontech).

Human embryo and fetus sections were obtained by the Joint MRC/Wellcome Trust (Grant #099175/Z/12/Z) Human Developmental Biology Resource (www.hdbr.org) with full ethical approval from the Joint Great Ormond Street Hospital National Health Service Trust/Institute of

Child Health Ethics Committee. In situ hybridization was carried out as previously described (Wilkinson 1998). Paraffin sections of embryos were dewaxed with Histo-clear and hydrated in ethanol gradients. Sections were washed twice in Phosphate buffered saline (PBS) and fixed in 4% paraformaldehyde (Sigma). Sections were washed twice in PBS and then incubated with proteinase K (Sigma) followed by fixation in 4% PFA. After two washes with PBS, sections were treated with triethanolamine and 0.25% acetic anhydride, and washed again twice in PBS. Slides were dehydrated and air dried. 300ng of digoxigenin-labelled antisense probe per slide was added to the hybridization mix and incubated on the sections overnight at 65°C in a humid chamber. Post hybridization washes were performed at 65°C for 20 minutes in the following order: 2x SSC, 2x formamide, 2x SSC, and 0.2x SSC. After cooling to room temperature, slides were incubated for 1 hour in blocking solution and finally incubated with anti-digoxigenin alkaline phosphatase-conjugated antibody solution overnight (Roche). Expression was visualized using NBT/BCIP (Roche). Sections were mounted using Vectamount (Vector Laboratories) and imaged with the Axioplan2 imaging system (Zeiss). Specificity of the in situ hybridization was confirmed with sense probes, which showed minimal signal.

Knockout mice

Mice heterozygous for a *Donson* knockout allele were produced as part of the European Conditional Mouse Mutagenesis Program (EUCOMM) and the International Knockout Mouse Consortium and phenotyped at the Wellcome Trust Sanger Institute. The *Donson*$^{tm1a(EUCOMM)Wtsi}$ knockout allele is a promoterless lacZ gene-trap cassette (Skarnes et al. 2011) inserted into intron 3 of *Donson* in C57BL/6N embryonic stem cells. This produces a null allele by splicing to the lacZ cassette, which contains an En2 splice acceptor and an SV40 polyadenylation sequence (Skarnes et al. 2011). Heterozygous mice were mated and offspring pups were genotyped (**Table 2**). Phenotyping was performed as part of the Sanger Institute Mouse Genetics Project and the International Mouse Phenotyping Consortium (MPC) (White et al. 2013). See the IMPC website (https://www.mousephenotype.org) for a listing of the 159 anatomic and laboratory phenotypes assayed in *Donson* heterozygous knockout mice. Only 1 of the 159 assayed phenotypes (mean red blood cell hemoglobin concentration) had a minor difference (small increase) in knockout mice that reached statistical significance.

siRNA knockdown and qPCR

HeLa (HLR-CHOP) cells (Stratagene) were grown in DMEM supplemented with 10% FBS, 1% L-glutamine, 1% penicillin/streptomycin, hygromycin, and G418. Cells were transfected with 40 nM siRNA targeted to *DONSON* or GFP, and 48 hours after transfection cells were stimulated with TNF-α (33 ng/ml). Total RNA was isolated from 10^7 cells in two biological replicate transfections per siRNA condition, and cDNA was synthesized using the Transcriptor First Strand Synthesis kit (Roche) with a mixture of oligo-dT and random hexamer primers. qPCR was performed with the RealTime ready Human Cell Cycle Regulation 91-gene Panel (Roche) and RealTime ready Human Reference Gene Panel (Roche) on a LightCycler 480 instrument with LightCycler 480 Probes Master solution. For each of the two biological replicate siRNA transfections, qPCR reactions of each gene/siRNA combination were run in triplicate. Relative gene expression between conditions (*DONSON* vs. GFP siRNA) was calculated after first normalizing each condition by the average expression level of a set of 19 housekeeping genes from the Reference Gene Panel using the ddCT method. This work was previously included in

the non-peer reviewed Biochemica newsletter of Roche (Zhang, et al. 2009. qPCR Identification of Genes Involved in Apoptosis and Cell Cycle Regulation. *Biochemica* **2**: 21-24).

qPCR of samples with the MMS variant and control samples (**Supplemental Fig. 2C**) was performed with a multiplex 18S rRNA (VIC-labeled, primer limited) and DONSON (FAM-labeled) TaqMan qPCR assay (Thermo Fisher) on a StepOnePlus Real-Time PCR System (Applied Biosystems). Each reaction contained 10 ul of 2x TaqMan Gene Expression Master Mix (Applied Biosystems), 1 ul each of the 18S and DONSON 20x TaqMan primer and probe mixes, cDNA, and water to a total volume of 20 ul. Each reaction was run in triplicate (technical replicates). Results were analyzed with StepOne Software v2.3 (Applied Biosystems).

Supplemental References

Altschul SF, Gish W, Miller W, Myers EW, Lipman DJ. 1990. Basic local alignment search tool. *J Mol Biol* **215**: 403-410.

Kent WJ, Sugnet CW, Furey TS, Roskin KM, Pringle TH, Zahler AM, Haussler D. 2002. The human genome browser at UCSC. *Genome Res* **12**: 996-1006.

Pertea M, Kim D, Pertea GM, Leek JT, Salzberg SL. 2016. Transcript-level expression analysis of RNA-seq experiments with HISAT, StringTie and Ballgown. *Nat Protoc* **11**: 1650-1667.

Pollard KS, Hubisz MJ, Rosenbloom KR, Siepel A. 2010. Detection of nonneutral substitution rates on mammalian phylogenies. *Genome Res* **20**: 110-121.

Siepel A, Bejerano G, Pedersen JS, Hinrichs AS, Hou M, Rosenbloom K, Clawson H, Spieth J, Hillier LW, Richards S et al. 2005. Evolutionarily conserved elements in vertebrate, insect, worm, and yeast genomes. *Genome Res* **15**: 1034-1050.

Skarnes WC, Rosen B, West AP, Koutsourakis M, Bushell W, Iyer V, Mujica AO, Thomas M, Harrow J, Cox T et al. 2011. A conditional knockout resource for the genome-wide study of mouse gene function. *Nature* **474**: 337-342.

Visel A, Thaller C, Eichele G. 2004. GenePaint.org: an atlas of gene expression patterns in the mouse embryo. *Nucleic Acids Res* **32**: D552-556.

Wilkinson DG. 1998. *In situ hybridization: a practical approach.* Oxford University Press, Oxford ; New York.
